# ZOLA-3D allows flexible 3D localization microscopy over an adjustable axial range

**DOI:** 10.1038/s41467-018-04709-4

**Published:** 2018-06-19

**Authors:** Andrey Aristov, Benoit Lelandais, Elena Rensen, Christophe Zimmer

**Affiliations:** 1 Unité Imagerie et Modélisation, Institut Pasteur, 25-28 rue du Docteur Roux, Paris, France; 2 UMR 3691, CNRS; C3BI, USR 3756, IP CNRS, Paris, France; 3 Hub Bioinformatique et Biostatistique, Institut Pasteur, Paris, France

## Abstract

Single molecule localization microscopy can generate 3D super-resolution images without scanning by leveraging the axial variations of normal or engineered point spread functions (PSF). Successful implementation of these approaches for extended axial ranges remains, however, challenging. We present Zernike Optimized Localization Approach in 3D (ZOLA-3D), an easy-to-use computational and optical solution that achieves optimal resolution over a tunable axial range. We use ZOLA-3D to demonstrate 3D super-resolution imaging of mitochondria, nuclear pores and microtubules in entire nuclei or cells up to ~5 μm deep.

## Abstract (web summary)

3D single-molecule localization is limited in depth and often requires using a wide range of point spread functions (PSFs). Here the authors present an optical solution featuring a deformable mirror to generate different PSFs and easy-to-use software for super-resolution imaging up to 5 µm deep.

## Introduction

Single molecule localization microscopy (SMLM) has arguably become the most popular approach to super-resolution fluorescence microscopy. Ever since its original demonstration for 2D imaging^1,2^, efforts have been made to extend this technique to 3D imaging^3–13^. Among the most widely used methods for 3D SMLM are those that exploit the axial variation of the point spread function (PSF) to determine the three spatial coordinates (*x*, *y*, *z*) of individual molecules from single 2D images^3–5,8,12,14,15^. Although under certain conditions it might be possible to obtain some 3D information over a limited (e.g., ~ 600 nm) axial range using a standard PSF^14,16,17^ (up to 1.4 μm in a recent report^18^), considerably larger axial ranges can be reached with optically engineered PSFs. The simplest and most common PSF engineering uses a cylindrical lens to induce astigmatism, achieving a *z*-range of ~ 1 μm^3,19^. A larger axial range (~ 2–3 μm) is possible with more complex PSFs such as a double helix^5^. Initial implementations using spatial light modulators (SLMs) based on liquid crystals enable rapid, software-controlled, deformations of the PSF, but entail a considerable loss of photons and hence resolution^7,20^. Transmission masks can be fabricated to generate specific PSFs, but are costly and cannot be adjusted to ensure optimal trade-offs between resolution and axial range for different samples. A promising alternative is provided by deformable mirrors (DMs) owing to their low cost and high photon efficiency. These devices are well suited to generate a wide range of PSFs, with very different shapes, lateral and axial extensions. Although several reconstruction algorithms have been developed for 3D SMLM, only few can handle a variety of engineered PSFs^8,12,15,20,21^. Among those, algorithms based on ad-hoc interpolation functions require extensive calibration data to achieve optimal precision, while those based on optical models rarely adopt an optimal PSF estimation strategy that takes into account the Poisson statistics of the images^22^. Furthermore, to the best of our knowledge, a complete user-friendly and free software for 3D SMLM image reconstruction with flexible PSFs is still lacking. Here, we describe Zernike Optimized Localization Approach in 3D (ZOLA-3D, or simply ZOLA), an integrated computational and optical strategy that offers an affordable, easy-to-use and versatile tool for 3D SMLM. We demonstrate ZOLA by imaging mitochondria, nuclear pores and microtubules over multiple micrometer depth, with water or oil immersion objectives, and with optimal 3D resolution.

## Results

### ZOLA-3D optical system and software

**Fig. 1:**
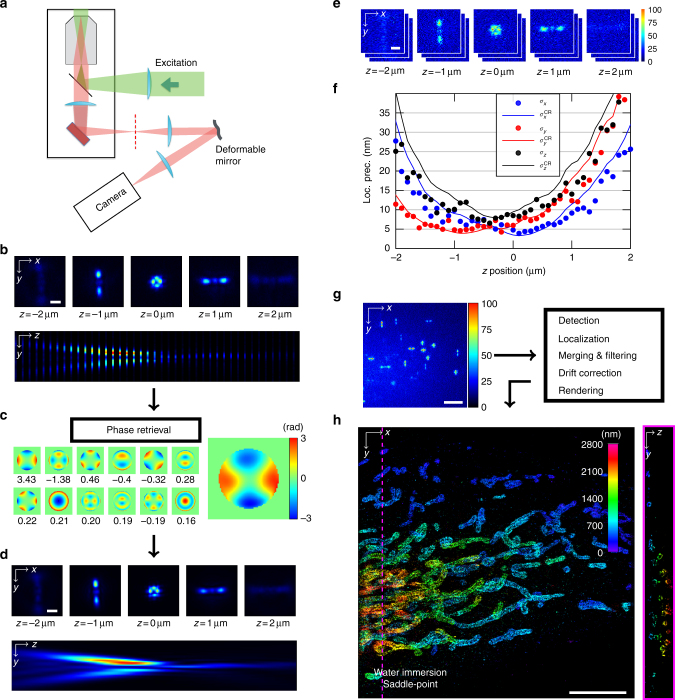
ZOLA-3D optical setup and algorithm. **a** Sketch of the optical system, featuring the objective lens, the deformable mirror and the camera. The deformable mirror is placed in the Fourier plane of the emission light path; its shape is controlled by 40 actuators (see Supplementary Fig. 1). **b**–**d** PSF calibration from bead images. Scale bars, 1 μm. **b** A *z*-stack of one or more subdiffraction sized fluorescent beads is acquired. Here, 40 images are taken over an axial range of 4 μm, with Δ*z* = 100 nm steps. Top row shows one image for every 1 μm step, the bottom row shows a (*y*, *z*) slice of the entire stack. **c** ZOLA uses these images to compute a maximum likelihood estimation of the phase (shown on the right, with color indicating phase) as a linear combination of Zernike polynomials (the twelve Zernike functions with the highest calculated coefficients are shown as small images, with their coefficients beneath). **d** PSF model computed by ZOLA from the retrieved phase, shown as in **b**. Unlike the 3D bead image, the PSF model is continuous, i.e., can be computed for any subpixelic position. **e** Fifty images of a fluorescent bead are taken at each of 40 *z* positions (with Δ*z* = 100 nm, i.e., over 4 μm). Scale bar, 1 μm. **f** Localization precisions as a function of *z*. Blue, red and black dots are average experimental localization precisions, defined as standard deviations of computed coordinates *x*, *y*, and *z*, respectively. Solid curves are theoretical precision limits, assuming a mean photon number of 4677 and mean background of 18.2 as in the bead images. **g** A single molecule image sequence is processed by ZOLA (a single frame is shown; scale bar, 5 μm). Processing includes detection, localization, merging consecutive localizations, filtering, drift correction, and super-resolution image rendering. **h** 3D super-resolution image of the mitochondrial protein TOM22 in a Cos7 cell, with color indicating depth *z*. Scale bar, 5 μm. The right panel shows a (*y*, *z*) slice at the position indicated by the pink dashed line. Supplementary Movie 2 shows an animated 3D view

Added to a standard SMLM system, our setup features a DM placed in the Fourier plane of the microscope, which allows one to spatially modulate the phase of the fluorescent signal by means of 40 independently controllable actuators (Fig. 1a and Supplementary Fig. 1). This system can generate very different PSFs, including but not limited to astigmatism^3,19^ and the more recently proposed saddle point or tetrapod PSFs (Fig. 1b and Supplementary Fig. 2), which have been shown to allow optimal resolution for specific desired axial ranges^20,22^. Computing precise (*x*, *y*, *z*) molecular coordinates from these images require a realistic model of the PSF that accounts for optical aberrations, which can be particularly severe for PSFs designed to capture larger axial ranges when using oil immersion optics^21–23^. In ZOLA, we therefore implemented a phase retrieval method^21^ to determine a realistic PSF model from a *z*-stack of subdiffraction fluorescent beads (Fig. 1b). Our method models the phase using Zernike polynomials^22^ and unlike the popular Gerchberg–Saxton algorithm^24^ uses a maximum likelihood estimation (MLE) algorithm that fully accounts for Poisson noise and is applicable to both EMCCD and sCMOS cameras. The Zernike coefficients (Fig. 1c) and additional parameters retrieved by ZOLA allow one to numerically predict the PSF for arbitrary subpixelic (*x*, *y*, *z*) positions (Fig. 1d), as required to determine precise localizations of single molecules. Our phase-retrieved PSF model provides an excellent match to the experimental PSF for a variety of engineered shapes (Fig. 1b, d). Compared to interpolation approaches such as cubic splines^18,25^, ZOLA requires far fewer calibration images for accurate PSF modeling (typically only one to four fluorescent beads are sufficient) and, importantly, can model spherical aberrations induced by refractive index mismatch between sample mounting medium and immersion medium using only beads on the coverslip^21^ (Supplementary Fig. 3). Traditional methods for localizing single molecules, such as fitting Gaussians to intensity peaks, are ill suited to spatially extended and multi-lobed PSFs such as saddle point or tetrapod PSFs. Instead, ZOLA uses the model PSF for pixel-level detection of single molecules via 3D cross-correlations and for rapid, subdiffraction precision localization via another MLE algorithm. ZOLA also features a tool for 3D drift correction based on redundant cross-correlations that does not require fiducial markers^26^ as well as a tool to merge consecutive localizations of the same molecule. In addition, ZOLA computes the fundamental limit to localization precision (Cramér–Rao lower bound (CRLB)^27^) based on the retrieved PSF and measured photon counts for each molecule (Fig. 1f and Supplementary Fig. 2), allowing to determine the theoretical resolution limit and to filter out low precision localizations. Furthermore, ZOLA provides a feature to automatically determine the Zernike coefficients (and hence the PSF) that achieve the lowest average CRLB over any specified axial range^20^ (Supplementary Fig. 4). This removes the need to test multiple, potentially sub-optimal PSFs, and instead enables the user to define DM settings that optimize the trade-off between axial range and resolution^20^. ZOLA is available as a plugin to the widely employed ImageJ/Fiji platforms^28^ and runs on CPUs or GPUs (graphical processing units; recommended for faster processing).

### Super-resolution 3D imaging over 2–5 μm depth

To assess ZOLA’s performance, we first measured the localization precision by repeated imaging of weakly excited fluorescent beads (Fig. 1e). The experimentally measured precision was very close to the CRLB, thus indicating optimal precision, as shown for a saddle point PSF in Fig. 1f (see Supplementary Fig. 5 for results on simulated images and for an astigmatic PSF, where a widely employed Gaussian fitting algorithm^29^ achieved less good axial precision and suffered from strong bias outside a range of ~ 1 μm). To demonstrate ZOLA's applicability to 3D super-resolution imaging, we first used a saddle point PSF and a water immersion objective to image mitochondria in Cos7 cells by immunolabeling of the protein TOM22, a component of the translocase complex of the outer mitochondrial membrane. We obtained 81,578 diffraction limited frames (Fig. 1g and Supplementary Movie 1) and processed them using ZOLA in about 2 h, resulting in *n* ≈ 0.96 million localizations (*n* ≈ 0.21 million after merging and filtering). Figure 1h shows a reconstructed 3D super-resolution image, with color indicating *z*. In both horizontal and vertical sections, the tubular structure of mitochondria is readily apparent. Supplementary Movie 2 shows several cuts through the 3D volume. The image covers an axial range of ~ 3 μm with an estimated resolution of ≈32–40 nm laterally and ≈36 nm axially (Supplementary Fig. 6a). This example illustrates the ability of our method to obtain high quality 3D super-resolution images over several micrometers axial range in reasonable time.

**Fig. 2:**
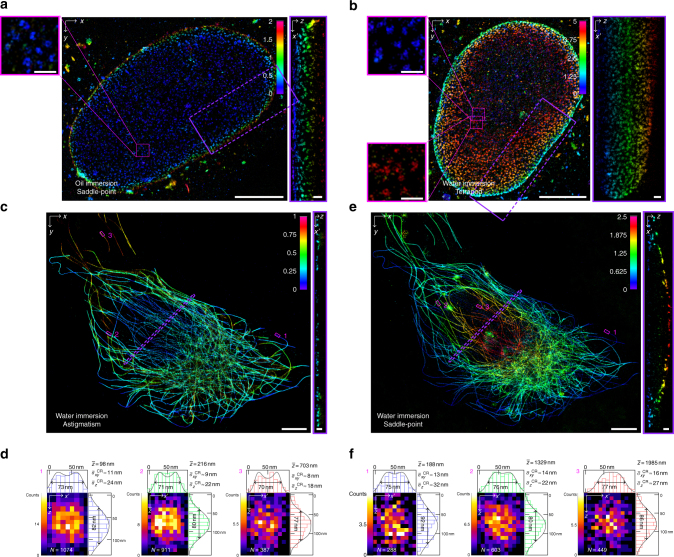
Three-dimensional super-resolution imaging over an adjustable axial range. **a**, **b** 3D super-resolution images of the nucleoporin Nup133 in HeLa cells reconstructed by ZOLA for a saddle point PSF with oil immersion objective and a tetrapod PSF with water immersion objective, respectively. Color indicates depth *z*. The axial range is 2 μm in **a**, showing the bottom portion of the nucleus, and 5 μm in **b**, allowing to visualize almost the entire nucleus. The (*x*′, *z*) view shows a projection from the region of interest enclosed by the violet dashed rectangle. Magnified views of pink boxed regions show nuclear pores visible as ring-like structures. Scale bars are 5 μm for the main images, and 0.5 μm for insets and (*x*′, *z*) projections. **c**, **e** 3D super-resolution images of microtubules in a U-373 MG cell. The same cell was imaged first with an astigmatic PSF (**c**), then with a saddle point PSF (**e**). The astigmatic PSF enables an axial range of 1 μm, allowing to visualize the bottom of the cell. The saddle point PSF enables an axial range of 2.5 μm, allowing to visualize the full cell. The (*x*′, *z*) view shows a projection from the region of interest enclosed by the violet dashed rectangle. Scale bars are 5 μm for the main images, 0.5 μm for insets and (*x*′, *z*) projections. **d**, **f** Histograms (2D and 1D) show the distribution of lateral and axial (*z*) coordinates of localizations across microtubule filaments at the three positions indicated by the pink rectangles in images **c** and **e** above. The number of localizations (*N*) and the mean *z*-coordinate ($\bar z_{}^{}$) are indicated. Black curves show the probability densities of axial and lateral coordinates expected for optimal precision, based on the average theoretical precision limits (Cramér–Rao lower bounds $\bar \sigma _{xy}^{\mathrm {CR}}$ and $\bar \sigma _z^{\mathrm {CR}}$ of lateral and axial localization errors are indicated) and the diameter of immunolabeled tubulin filaments (see Supplementary Fig. 8). Full width at half maxima of the probability densities are indicated below double arrows. The good match between the theoretical probability densities and the experimental histograms indicates that ZOLA achieves optimal precision at all depths

To demonstrate the versatility of ZOLA, we next imaged immunolabeled Nup133 nucleoporins in HeLa cells. Using a saddle point PSF with an oil immersion objective we acquired 50,000 frames, resulting in *n* ≈ 0.40 million localizations (*n* ≈ 0.25 million after merging and filtering). The reconstructed 3D image had a resolution of ≈42–54 nm laterally and ≈61 nm axially (Supplementary Fig. 6b), allowing to visualize nuclear pores as rings of 100.2 ± 9.7 nm diameter (mean ± std.dev. for 10 rings) (Fig. 2a). The axial range was ~ 2 μm, limited by spherical aberrations due to refractive index mismatch. However, using a water immersion objective with a tetrapod PSF enabled us to visualize almost the entire nuclear envelope over an axial range of ~ 5 μm (Fig. 2b) (image reconstructed from 50,000 frames and *n* ≈ 0.49 million localizations, *n* ≈ 0.24 million after merging and filtering) (see also Supplementary Movie 3). Although the resolution was slightly reduced to ≈54–57 nm laterally and ≈74 nm axially (Supplementary Fig. 6c), it still allowed to distinguish the ring-like structure of nuclear pores. Supplementary Fig. 7 shows another example of an entire nucleus imaged over ~ 4.5 μm depth.

An unavoidable drawback of PSFs engineered to capture a large axial range, such as saddle point or tetrapod PSFs, is that their larger lateral extent increases fluorescence overlaps from nearby molecules, preventing their precise localization. Although strong pre-bleaching can reduce the density of activated fluorophores, this delays productive imaging by up to several minutes and depletes the sample of potentially informative fluorescence^30^. An alternative is to use a more compact PSF, like the astigmatic PSF, at the beginning of image acquisition, when activation density is high, and switch to a more extended PSF once activation density becomes low enough to avoid overlaps, thereby minimizing information loss due to pre-bleaching. We took advantage of the flexibility of our DM system to demonstrate this, using alternating DM settings to image a single U-373 cell with immunolabeled microtubules. We first applied an astigmatic PSF for 46,621 frames, and then a saddle point PSF for 87,959 frames using water immersion optics (Supplementary Movie 4). ZOLA analysis resulted in *n* ≈ 2.5 and 2.9 million localizations (*n* ≈ 0.8 and 1.4 million after merging and filtering), with processing times of about 4 and 8 h, respectively. Figure 2c shows the 3D image obtained with astigmatism, which shows the bottom of the cell over an axial range of ~ 1 μm. Histograms of localizations across individual filaments at three axial positions (z = 98, 216 and 703 nm) are consistent with the theoretical precision limit computed by ZOLA -considering the ~ 25 nm diameter of microtubules and antibody size- and indicate resolutions of ≈18–25 nm laterally and ≈41–55 nm axially (Fig. 2d and Supplementary Fig. 8). Figure 2e shows the 3D image of the same cell obtained with a saddle point PSF, which covers a larger axial range of ~ 2.5 μm and encompasses the entire cell. Localization histograms across filaments (at *z* = 188, 1329, and 1985 nm), again agree with predicted resolution limits, namely ≈29–37 nm laterally and ≈51–74 nm axially (Fig. 2f). Supplementary Fig. 9 shows another example. Comparing images obtained with the astigmatic, saddle point and tetrapod PSFs illustrates the trade-off between axial range and spatial resolution as well as the flexibility of our DM-based imaging system.

## Discussion

In summary, ZOLA-3D is a versatile, easy-to-use optical and computational imaging system for 3D SMLM that achieves theoretically optimal resolution over adjustable axial ranges from <1 μm up to at least 5 μm. Even larger axial ranges can in principle be achieved using different PSFs, although their larger lateral extent implies that the density of active fluorophores must be kept very low to maintain high localization precision. Note also that ZOLA may be used to track single particles or molecules in 3D in live cells and that the use of reflective, rather than refractive optics will facilitate applications to multicolor imaging. Our software is freely available from https://github.com/imodpasteur/ZOLA-3D, together with sample data and instructions. We anticipate that ZOLA-3D will greatly facilitate 3D imaging of entire nuclei and cells with super-resolution.

## Methods

### Optical setup and deformable mirror

Our 3D localization microscopy system is based on an inverted microscope body (Nikon Eclipse Ti). We used either a 60× water immersion objective lens with numerical aperture 1.2 (Nikon, CFI Plan Apo VC 60XC WI) or a 60× oil immersion objective lens with numerical aperture 1.49 (Nikon, CFI Apo TIRF 60X Oil), staged on a z-piezo module (Physik Instrumente GmbH). Widefield illumination was achieved with a 500-mW 642 nm laser (MPB Communications Inc.). Laser power was modulated with an acousto-optic tunable filter (AOTF, AA Optics) placed on a pneumatically stabilized optical table. Images were formed through the right port of the microscope via a 4F image-relay system that includes F100 and F200 mm lenses (Thorlabs). A deformable mirror (DMP40-P01, Thorlabs) was placed on a manual XY stage in the Fourier plane of the 4F system. Images were collected with an EMCCD camera (Andor iXon Ultra) controlled by the Micromanager software^31^. The measured pixel size of the camera was 110 nm, in a good agreement with the 108.3 nm size expected from the physical camera pixel size of 13 μm, the 60× objective lens and the 2× zoom in the image relay system. We used Nikon’s perfect focus system to maintain focus. In order to minimize drift, the entire setup was shielded from air flux and the temperature of the room was maintained stable within 1 °C. Residual axial and lateral drift were corrected after image acquisition using ZOLA’s drift correction feature (see section Drift correction below).

### Deformable mirror setting

We used the native software supplied with the DM to control each of 40 segments of the mirror. Note that even when applied voltages are set to zero the DM is not perfectly flat but contains minor aberrations of the reflective surface leading to second- and third-order deformations of the PSF. We therefore first corrected these primary aberrations by adjusting the Zernike modes of the DM, the collar ring of the objective lens and the lateral position of the DM in order to maximize the symmetry and brightness of the PSF as determined from images of 0.1 μm diameter beads (Tetraspeck). After this correction, we applied voltage settings to create the specific engineered PSFs mentioned in the main text. For low depth imaging (Fig. 2c), astigmatism was created using pre-specified software settings, with the amount of deformation set manually. For imaging with extended axial range (Figs. 1h and 2a, b, e), voltages were applied manually to create saddle-point or tetrapod PSFs. PSFs were optimized either by visual inspection or on the basis of the theoretical precision limit (CRLB) computed by ZOLA after phase retrieval (see section PSF optimization below). All PSF-calibration stacks were recorded prior to single-molecule imaging in the same sample using fluorescent beads close to the cells of interest.

### Image formation model

ZOLA's PSF modeling algorithm takes as input a *z*-stack of one or more fluorescent beads and outputs a PSF model. After selection of *S* beads by the user, an equal number of *z*-stacks is created by defining regions of interest around each bead. PSF modeling is achieved via a MLE algorithm described below (section PSF calibration by phase retrieval), which is based on the following image formation model:

1 $${\cal M}_{s,k,i} = A_{s,k}{\cal P}_i\left( {{\mathrm{d}}x_s,{\mathrm{d}}y_s,z_k + {\mathrm{d}}z_s} \right) + B_{s,i}.$$

Here, ${\cal M}_{s,k,i}$ is the image model intensity at the pixel of index *i* in frame *k* ∈ [1, *K*] of the *z*-stack containing bead *s* ∈ [1, *S*]; *A*_*s*,*k*_ is the number of photons associated with bead *s* in this frame (we allow this number to depend on *k* in order to account for photobleaching during *z*-stack acquisition). ${\cal P}_i(x,y,z)$ is the intensity at pixel *i* of the PSF, i.e., the image of a point light source at position (*x*, *y*, *z*); (d*x*_*s*_, d*y*_*s*_, d*z*_*s*_) designates the 3D position of each bead, and *z*_*k*_ is the distance of the imaged focal plane relative to the coverslip. *B*_*s*,*i*_ is the number of background photons at pixel *i*.

Assuming that the image follows Poisson statistics, the probability of observing a given stack of images ${\cal I}_{s,k,i}$ (*i* = 1…*N*, *k* = 1…*K*, *s* = 1…*S*) can be written:

2 $$p({\cal I}|{\cal M}) = \mathop {\prod}\limits_{s = 1}^S \mathop {\prod}\limits_{k = 1}^K \mathop {\prod}\limits_{i = 1}^N \frac{{{\cal M}_{s,k,i}^{{\cal I}_{s,k,i}}{\mathrm{e}}^{ - {\cal M}_{s,k,i}}}}{{{\cal I}_{s,k,i}!}}.$$

This noise model is suited for EMCCD cameras. For sCMOS cameras with pixel-dependent gain, offset and readout noise, the statistical model was modified as in ref ^32^. A point source of light at position (*x*, *y*, *z*) in the object domain should lead to the following distribution of fluorescence in the image domain^33^:

3 $$\begin{array}{l}{\cal P}_i^{{\mathrm{th}}}({\mathrm{\Phi }},x,y,z)\\ = \left| {{\cal F}_{k_x,k_y}\left( {\rho \left( {k_x,k_y} \right){\mathrm{e}}^{ {{{j\Phi }}(k_x,k_y)} }{\mathrm{e}}^{ {2 j\pi (xk_x + yk_y)} }{\mathrm{e}}^{{2j\pi (k_{z_2}z - k_{z_1}f)} }} \right)} \right|^2 \;\;\; i \in \left[ {1,N} \right]\end{array}.$$

Here *N* is the number of pixels, *i* the pixel index, *ρ*(*k*_*x*_, *k*_*y*_) is the indicator function of the disk of radius NA/*λ* (i.e., *ρ*(*k*_*x*_, *k*_*y*_) = 1 for (*k*_*x*_ + *k*_*y*_)^2^ ≤ (NA/*λ*)^2^ and *ρ*(*k*_*x*_, *k*_*y*_) = 0 elsewhere), NA is the numerical aperture of the objective lens and *λ* the emission wavelength of the dye, *j* is the unit imaginary number (*j*^2^ = −1), Φ is the phase mask at the pupil plane, *f* is the distance between the coverslip surface and the position of the perfect focus and $k_{z_{\{ 1,2\} }}$ = $\sqrt {\left( {\frac{{n_{\{ 1,2\} }}}{{\mathit \lambda}}} \right)^2 - \left( {k_x^2 + k_y^2} \right)}$, where *n*_1_ and *n*_2_ are the refractive indices of the immersion medium and the sample, respectively. ${\cal F}$ denotes the Fourier transform operator.

In practice, the image of a fluorescent bead is not as sharp as predicted by Eq. (3) due to aberrations not accounted for by the model (Supplementary Figs. 10 and 11). To address this, we introduce a convolution with a Gaussian kernel as follows^21^:

4 $${\cal P}_i({\mathrm{\Phi }},x,y,z,\sigma ) = {\cal P}_i^{{\mathrm{th}}}({\mathrm{\Phi }},x,y,z) \otimes G(\sigma ),$$

where *G*(*σ*) is a two-dimensional Gaussian function of standard deviation *σ* and ⊗ is the convolution operator. We empirically set the parameter *σ* to 0.7 pixels in all our experiments, which allowed a good match to the experimental PSF (Supplementary Fig. 10). However, to provide a more general solution, the ZOLA plugin estimates the optimal value of *σ* during phase retrieval along with the Zernike coefficients (see section PSF calibration by phase retrieval below). This allows ZOLA to correctly model aberrated PSFs on different microscope setups (Supplementary Fig. 11).

### Modeling of phase and background

For efficient phase retrieval, we assume that the phase can be represented by a combination of *J* − 3 Zernike polynomials^22^ (shown in Supplementary Fig. 4f):

5 $${\mathrm{\Phi }}\left( {k_x,k_y} \right) = \mathop {\sum}\limits_{j = 4}^J {\kern 1pt} c_j{\cal Z}_j\left( {k_x,k_y} \right),$$

where ${\cal Z}_j$ is the Zernike polynomial for index *j*. The three first Zernike polynomials (called piston, tip and tilt) do not influence the shape of the PSF and are therefore not taken into account. The advantage of using a limited set of Zernike polynomials to model the phase (rather than a phase image) is that relatively few parameters need to be estimated. This model is very flexible, except for modeling phase jumps (as in the double helix PSF^5^), and provides robustness to image noise. This is very important, because *z*-stack acquisition must be fast to avoid spatial drift from affecting PSF modeling, which results in images with limited signal-to-noise ratio. Furthermore, robustness to noise means that ZOLA requires very few beads for accurate PSF modeling. For the results shown in this paper, we used only one calibration bead per imaging experiment, as shown in Fig. 1, but in general we recommend using multiple (e.g., 2–3) beads, especially if the photon counts per bead is less than ~4000 (see Supplementary Fig. 12a). We used *J* = 36 in our experiments, which is more than sufficient to model many complex PSFs (Supplementary Fig. 12b, c). If bead images have low signal-to-noise ratio, one can increase the number of beads and decrease *J*. Thus, our phase modeling approach limits the detrimental effect of drift and does not necessitate extensive calibration data.

In addition to a model of the PSF, detailed above (Eqs. (3) and (4)), our image formation model (Eq. (1)) requires a model of the local background *B*_*s*,*i*_. Because of potentially nonuniform laser illumination, we allow the background to vary laterally within the field of view. This is important because the region of interest needs to be large in order to encompass optically engineered PSF shapes. We assume that the background can be approximated by a two-dimensional second-order polynomial function, defined by six parameters:

6 $$B_{s,i} = B_s^0{{x}}^2 + B_s^1{{y}}^2 + B_s^2{{xy}} + B_s^3{{x}} + B_s^4{{y}} + B_s^5,$$

where (*x*, *y*) is the position of the pixel *i*.

### PSF calibration by phase retrieval

From Eqs. (3)–(6), the set of parameters that fully determines the model image (Eq. (1)) is

7 $${\mathrm{\Theta }} = \left\{ {c_4, \ldots ,c_J,\theta _1, \ldots ,\theta _S,\sigma } \right\}$$

with

8 $$\theta _s = \left\{ {A_{s,1}, \ldots ,A_{s,K},B_s^0, \ldots ,B_s^5,{\mathrm{d}}x_s,{\mathrm{d}}y_s,{\mathrm{d}}z_s} \right\} \;\;\;s \in \left[ {1,S} \right]$$

and the model image can be written as a function of the parameters as

9 $${\cal M}_{s,k,i}({\mathrm{\Theta }}) = \sum\limits _{s = 1}^S{\kern 1pt} A_{s,k}{\cal P}_i\left( {{\mathrm{\Phi }}({\mathrm{\Theta }}),{\mathrm{d}}x_s,{\mathrm{d}}y_s,z_k + {\mathrm{d}}z_s,\sigma } \right) + B_{s,i}({\mathrm{\Theta }})$$

where the PSF ${\cal P}$, phase Φ and background *B*_*s*,*i*_ are related to the parameters Θ as specified by Eqs. (3)–(6).

ZOLA’s phase retrieval algorithm estimates all these parameters from the *z*-stack by using MLE based on the Poisson noise model (Eq. (2)), i.e., by minimizing the negative log likelihood:

10 $${\hat{\mathrm \Theta }} = {\mathrm{arg}}\mathop {{{\mathrm{min}}}}\limits_{\mathrm{\Theta }} \left( { - {\mathrm{log}}{\kern 1pt} {\cal L}({\mathrm{\Theta }})} \right)$$

where

11 $${\cal L}({\mathrm{\Theta }}) = \mathop {\prod}\limits_{s = 1}^S \mathop {\prod}\limits_{k = 1}^K \mathop {\prod}\limits_{i = 1}^N \frac{{{\cal M}_{s,k,i}({\mathrm{\Theta }})^{{\cal I}_{s,k,i}}{\mathrm{e}}^{ - {\cal M}_{s,k,i}({\mathrm{\Theta }})}}}{{{\cal I}_{s,k,i}!}}$$

In practice, this optimization is performed by iteratively updating each scalar parameter using the Newton–Raphson algorithm^34^. We typically use 30 iterations. As shown in Fig. 1, our phase retrieval generates an accurate PSF model for any *x*, *y*, *z* positions.

### Single molecule detection

Single molecule localization in ZOLA is performed in two steps. First, single molecules are detected in 3D and localized with pixel-level precision. Second, a MLE algorithm is used to refine localizations with sub-pixelic and sub-diffraction precision. Because our engineered PSF can consist of multiple lobes (see, e.g., the saddle point PSF in Fig. 1b), detection cannot rely on searching local intensity maxima as commonly done for conventional PSFs. In order to detect single molecules with arbitrary PSFs, we use a 3D matched filtering approach, i.e., we compute 3D cross-correlation images between the observed image and the model PSF. More specifically, we first create a series of model PSF images for different axial positions using Eq. (4) and setting *x* = 0, *y* = 0, and *z* = {*z*_min_, *z*_min_ + *δz*, …, *z*_max_}, where *z*_min_ and *z*_max_ define the axial range of the PSF and *δz* the axial precision of the detection. Usually, we set *δz* equal to the size of the 2D pixels of the raw images (in our case 110 nm). Then, a 3D image is created by computing the normalized cross-correlation between the 2D single molecule image ${\cal I}$ and each model PSF image. For faster processing, these cross-correlations are performed using fast Fourier transforms (FFTs). Finally, extraction of local maxima in the 3D cross-correlation image provides a pixel-level detection of single molecules. Local maxima whose cross-correlation value falls below 0.2 are rejected, whereas the remaining maxima are considered as detected molecules. These detected molecules are finally ordered by decreasing cross-correlation for sequential localization refinement (see section below).

### Precise localization of single molecules

Precise localization of single molecules is performed by fitting the 3D PSF model (obtained as described in section PSF calibration by phase retrieval) to the image region around each detected pixel (as described in the previous section). The model image at pixel *i* is given by:

12 $${\cal M}_i = A{\cal P}_i(x,y,z) + B$$

where *A* is the photon number, *B* is the background (here assumed to be locally uniform), and *x*, *y*, *z* are the coordinates of the molecule. Localization refinement consists in estimating the five parameters Θ′ = {*A*, *B*, *x*, *y*, *z*}. This is done by minimizing the negative log-likelihood, similarly as for PSF calibration in Eqs. (10) and (11). Note that the parameters that define the PSF (Zernike coefficients *c*_*i*_ and Gaussian kernel standard deviation *σ*) are not fitted during single molecule localization, but held fixed to the values determined during PSF calibration. We again use a Newton–Raphson iterative algorithm to estimate Θ′, using the results of the detection step described in the previous section for parameter initialization. This initialization makes optimization fast and prevents convergence to local minima. Iterations stop if the estimated position changes by less than 0.1 nm, which on average occurs with less than 10 iterations, or when reaching the maximum iteration number of 30. Note also that our localization method is currently not designed to handle overlapping PSFs, although we plan to add this capability in a future version of ZOLA.

### Theoretical limit to localization precision

For unbiased estimators (i.e., estimators which provide the correct value on average), the mean squared error between the estimated and true parameter values is fundamentally limited by the CRLB^17^:

13 $$\left\langle {(\hat \theta - \theta )^2} \right\rangle \ge {\mathrm{CRLB}}_\theta = I_\theta ^{ - 1},$$

where *I*_*θ*_ is the Fisher information matrix given by

14 $$\left[ {I_\theta } \right]_{u,v} = \mathop {\sum}\limits_i \frac{1}{{{\cal M}_i(\theta )}}\frac{{\partial {\cal M}_i(\theta )}}{{\partial \theta _u}}\frac{{\partial {\cal M}_i(\theta )}}{{\partial \theta _v}}.$$

Applied to the parameters *x*, *y*, *z* in Θ′, the CRLB provides a theoretical limit to the localization precision (and hence resolution), such that

15 $$\sigma \left( {\hat x - x} \right) = \sqrt {\left\langle {(\hat x - x)^2} \right\rangle } \ge \sqrt {{\mathrm{CRLB}}_x} = \sigma _x^{{\mathrm{CR}}}$$

and likewise for *y* and *z*.

ZOLA provides a tool to calculate and plot $\sigma _x^{{\mathrm{CR}}}$, $\sigma _y^{{\mathrm{CR}}}$ and $\sigma _z^{{\mathrm{CR}}}$ for any phase-retrieved PSF and assumed photon numbers *A* and *B* for molecule and background, as a function of axial position *z*. We use this quantity to verify that ZOLA achieves optimal precision (Fig. 1f) and to optimize the PSF as detailed in the next section. In addition, ZOLA computes the CRLB for each individual molecular localization, providing a measure of its associated precision. The three CRLB values can therefore be used to filter out localizations with low precision (see section Localization filtering and merging).

### PSF optimization

ZOLA provides a feature to calculate the PSF that provides the best axial resolution over any specified axial range Δ*z*. Following ref. ^20^, we numerically determine the Zernike coefficients that achieve the lowest axial CRLB averaged over the axial range Δ*z*. We discretize the axial range as *z*_*i*_ = *z*_min_ + *iδz*, for *i* = 0, …, *k* − 1, where $\delta z = \frac{{{\mathrm{\Delta }}z}}{k}$, and *δz* is the axial step (we usually set $k=10$). We then define the PSF optimization problem as

16 $$\left( {c_4^ \ast , \ldots c_J^ \ast } \right) = {\mathrm{arg}}\mathop {{{\mathrm{min}}}}\limits_{\left( {c_4, \ldots c_J} \right)} \mathop {\sum}\limits_{i = 0}^k {\kern 1pt} \sigma _z^{{\mathrm{CR}}}\left( {z_i} \right)$$

where $\sigma _z^{{\mathrm{CR}}}\left( {z_i} \right)$ is the CRLB for the *z* coordinate at the axial position *z*_*i*_. This minimization is achieved iteratively using a Newton–Raphson algorithm as for PSF calibration and single molecule localization above. The CRLB also depends on the signal and background photon numbers *A* and *B*. For this optimization we set *A* = 3000 and *B* = 50 based on typical values in our experiments. Supplementary Fig. 4a–c shows PSFs and their corresponding phase optimized by ZOLA for axial ranges Δ*z* = 1 μm, Δ*z* = 3 μm, and Δ*z* = 5 μm, respectively. The resulting PSFs, respectively, resemble astigmatic, saddle point, and tetrapod PSFs^20^. Supplementary Fig. 4d shows the CRLBs for the three PSFs and the three coordinates. Supplementary Fig. 4e shows the Zernike coefficients of the optimized PSFs, with the Zernike polynomials shown in Supplementary Fig. 4f.

### Localization filtering and merging

As mentioned above, the CRLB values computed for each detected molecule provide a measure of the localization uncertainty and can be used to filter out the least precise localizations. For astigmatic PSFs, we removed localizations with lateral CRLB values ($\sigma _x^{{\mathrm{CR}}}$ and $\sigma _y^{{\mathrm{CR}}}$) ≥ 20 nm or axial CRLB ($\sigma _y^{{\mathrm{CR}}}$) ≥ 40 nm. For saddle point PSF, we removed localizations with $\sigma _x^{{\mathrm{CR}}}$ or $\sigma _y^{{\mathrm{CR}}}$ ≥ 30 nm or $\sigma _z^{{\mathrm{CR}}} \ge 60{\kern 1pt} {\mathrm{nm}}$. For tetrapod PSF, we did not apply this filtering.

The CRLB is based on the assumption that the data differs from the model only because of Poisson noise, which is not always true in practice, for example in the presence of nearby molecules with partly overlapping PSFs. We therefore also compute the *χ*^2^ score of the residual (the difference between the model and observed image) for each localization:

17 $$\chi ^2 = \mathop {\sum}\limits_i \frac{{\left( {{\cal I}_i - {\cal M}_i} \right)^2}}{{{\cal M}_i}}$$

A *χ*^2^ score close to 1 indicates that the noise obeys Poisson statistics (where variance equals mean) and thus that the model optimally fits the data. Localizations with high *χ*^2^ scores indicate the presence of signal or noise not accounted for by the PSF and Poisson noise models, which typically occurs due to proximity of other molecules. Filtering out localizations with high values of *χ*^2^ can therefore improve the quality and resolution of reconstructed images. We applied the filter *χ*^2^ ≤ 3, but more restrictive thresholds can be used for high density localization images.

In addition, ZOLA provides a feature to merge consecutive localizations that are highly likely to originate from the same molecule. The merging procedure depends on three parameters: (i) the lateral capture radius, (ii) the axial capture radius, (iii) the maximum number of frames the molecule is allowed to disappear before reappearing. In all our experiments, the lateral and axial capture radii were set to 100 nm and 200 nm, respectively. The maximum number of frames allowed for disappearance was set to 0 to prevent merging of different molecules. ZOLA merges consecutive localizations within the capture radii, i.e., replaces them with a single localization, which is assigned a number of signal photons equal to the sum of corresponding photon counts from the individual localizations. Background photon counts are averaged from individual localizations, and the coordinates of the resulting single localization is defined as the mean of individual positions weighted by their corresponding CRLB values.

### Drift correction

To create high quality super-resolution images, potential drift during image acquisition must be corrected. Correcting the drift in 3D in the absence of fiducial markers is challenging. In ZOLA, we achieve this using redundant cross-correlations. While this approach has been previously proposed for 2D drift correction^35^, we have extended it to 3D. The process consists of six steps^26^. In the first step, localizations are grouped within non-overlapping time windows, each consisting of typically 10,000 consecutive frames. A 3D super-resolution image is generated for each of these time windows (as a 3D histogram of localizations, with a specified 3D voxel size, see below). In the second step, we compute the 3D cross-correlation between all pairs of super-resolution images^35^ using 3D FFTs. In the third step, we estimate the 3D displacement for each pair of time windows by locating the maximum value of the corresponding 3D cross-correlation image. This is done with subvoxel precision by computing the weighted mean of voxel positions with intensities exceeding 95% of the maximum intensity. In the fourth step, for each time window, the 3D displacement is estimated from the displacements between all pairs of windows by least squares optimization^35^. In the fifth step, we fit a spline function to the estimated displacements to estimate the drift for each frame throughout the image sequence. In the sixth step, in each frame, the estimated displacement is subtracted from the computed molecule localizations.

Drift correction requires setting two parameters: the number of consecutive frames defining the non-overlapping time windows, and the size of 3D voxels in the reconstructed super-resolution images. We typically use time windows of 10,000 consecutive frames and voxel sizes of 30 × 30 × 30 nm. Setting smaller time windows and smaller voxels can in some cases improve the accuracy of drift correction, but considerably increases processing time and memory consumption. To limit memory usage, we split each reconstructed image in *M* overlapping 3D sub-images and compute *M* smaller cross-correlation images instead of one. By summing these *M* 3D cross-correlation images, we obtain the central part of the 3D cross-correlation (around zero displacement) that would have been obtained without splitting. The number *M* of 3D sub-images is determined by the voxel size above and the maximum expected drift, which constitutes an additional parameter. We usually set this parameter to 6 μm, which together with the voxel size above results in a memory usage of ~800 MB that is compatible with consumer grade GPU cards.

### Super-resolution image rendering

Super-resolution images are rendered as 2D or 3D average shifted histograms of 3D localizations. Color can be used to encode the axial coordinate to facilitate interpretation (see Figs. 1h and 2b, c, e). We also used the ViSP software to create animated 3D visualizations (Supplementary Movies 2 and 3)^36^. Users can import and export localization tables. For each localization, these tables indicate estimated position, photon number, background, *χ*^2^ of the residual, CRLB, and estimated drift (if applicable).

### Software and computing hardware

ZOLA-3D is provided as an ImageJ plugin available via the Fiji update system^28,37^. Instructions on how to obtain, install and use ZOLA-3D are available at https://github.com/imodpasteur/ZOLA-3D, along with sample data. We analyzed images using ZOLA on a Windows computer equipped with a Nvidia GTX480 GPU card, a Nvidia Quattro K4200 or on an Ubuntu machine with a Nvidia Tesla K40 GPU.

### Sample preparation

For microtubule imaging experiments (Fig. 2c, e), U-373 MG (Uppsala) cells from ATCC were cultured in Dulbecco' s Modified Eagle Medium: Nutrient Mixture F-12 (DMEM/F12; Gibco) supplemented with 10% (v/v) fetal bovine serum (FBS; Gibco), 1% (v/v) penicillin-streptomycin (Gibco), in a 5% CO_2_ environment at 37 °C on 18-mm cleaned coverslips in 12-well plates or 22-mm cleaned coverslips in 6-well plates. 24 h later, cells were pre-extracted for 10 s with 0.25% (v/v) Triton X-100 in BRB80 buffer (80 mM PIPES, 1 mM MgCl_2_, 1 mM EGTA, adjusted to pH 6.8 with KOH), and immediately fixed for 10 min with 0.25% (v/v) Triton X-100 + 0.5% (v/v) Glutaraldehyde in BRB80. Samples were incubated for 7 min with 0.1% NaBH_4_ solution in PBS and washed several times with PBS. Cells were directly incubated for 1 h at room temperature with a 1:500 dilution of rat anti-alpha-tubulin antibodies (Bio-Rad, ref. MCA77G) in PBS, washed three times with PBS, and then incubated for 45 min with a 1:500 dilution of anti-rat Alexa-647 conjugated secondary antibodies (Jackson ImmunoResearch Laboratories, ref. 712-605-153) in PBS.

For mitochondria imaging (Fig. 1h), COS7 cells from ATCC were cultured under the same conditions as U373 cells using phenol-red free DMEM medium and fixed with 4% (v/v) PFA in PBS for 10 min. The sample was blocked with 3% (w/v) BSA in PBS for 20 min and immunostained with a 1:500 dilution of anti-TOM22 antibodies (Sigma, ref. T6319) produced in mouse in wash buffer (0.5% BSA in PBS) for 1 h. After extensive washing with wash buffer, the sample was incubated with a 1:500 dilution of anti-mouse secondary antibodies produced in donkey conjugated with Alexa-647 (Jackson ImmunoResearch Laboratories, ref. 715-605-151) in wash buffer for 30 min. After washing five times with wash buffer and two times with PBS, samples were post-fixed with 2% (w/v) PFA in PBS for 10 min and washed five times with PBS.

For nuclear pore imaging (Fig. 2a, b), HeLa cells were cultured in phenol-red free DMEM medium. The samples were stained as the mitochondria samples above. The primary antibody, anti-Nup133 produced in rabbit (Abcam, ref. ab155990) was used at a 1:200 dilution and the secondary antibody conjugated with Alexa-647 produced in goat (Jackson ImmunoResearch Laboratories, ref. 111-605-003) was used at a 1:500 dilution. For all samples, we used a photoswitching buffer composed of 50 mM Tris-HCl + 10 mM NaCl + 10% (w/v) glucose + 168 AU/mL Glucose-Oxidase + 1404 AU/mL Catalase + 1% 2-Mercaptoethanol + 1% COT (cyclooctatetraene) adjusted to pH 8.

### Image acquisition

Single-molecule imaging of photoswitching fluorophores was performed using camera frame crop of 512 × 512 pixels for microtubules (Fig. 2c, e) and 256 × 256 pixels for mitochondria (Fig. 1h) and nuclear pores (Fig. 2a, b) with the camera gain set to 200 and using the photon counting mode. The power of the 642 nm laser was set to the maximum. Exposure time varied from 30 ms for the early stages of acquisition (to limit PSF overlaps) to 100 ms in the later stages of acquisition, when the density of activated fluorophores was low, to maximize signal to noise. We acquired 81,578 images for the mitochondria experiment (Fig. 1) and 50,000 images for each nuclear pore experiment (Fig. 2a, b). For microtubules, we acquired 46,621 images with astigmatism (Fig. 2c) and 87,959 frames using saddle-point PSF (Fig. 2e).

### Code availability

The ZOLA-3D ImageJ plugin and source code are freely available from Fiji and Github, respectively, as explained here: https://github.com/imodpasteur/ZOLA-3D

### Data availability

The localization data used to generate the super-resolution images in Figs. 1 and 2 are available from: https://github.com/imodpasteur/ZOLA-3D

## Electronic supplementary material

Supplementary Information

Description of Additional Supplementary Files

Supplementary Movie 1

Supplementary Movie 2

Supplementary Movie 3

Supplementary Movie 4
