# Supplementary material for: ZOLA-3D allows flexible 3D localization microscopy over an adjustable axial range
Source: Nat Commun. 2018 Jun 19;9:2409. doi: 10.1038/s41467-018-04709-4 (PMC6008307; doi:10.1038/s41467-018-04709-4)
Supplement: Supplementary file 1 — Supplementary Information [file 41467_2018_4709_MOESM1_ESM.pdf]

ZOLA-3D allows flexible 3D localization microscopy over an adjustable axial range

Aristov et al.

Supplementary Information

Contains Supplementary Figures 1-12 and Supplementary References

a

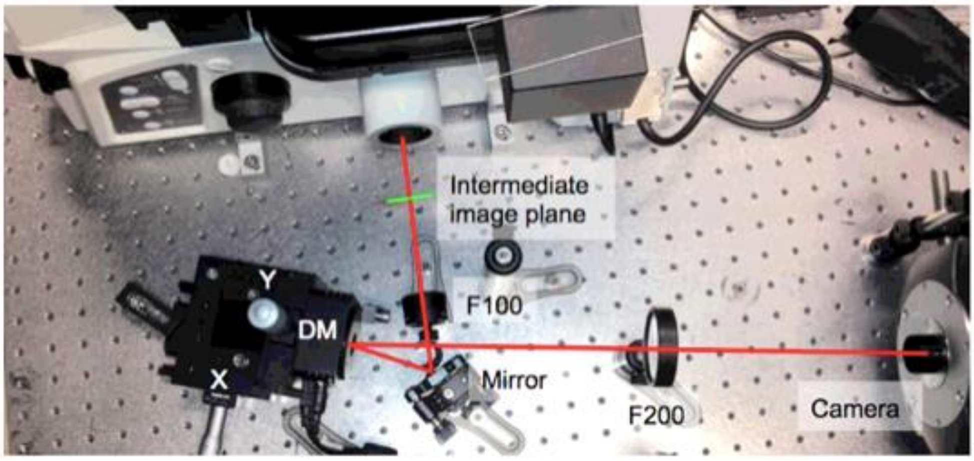

b

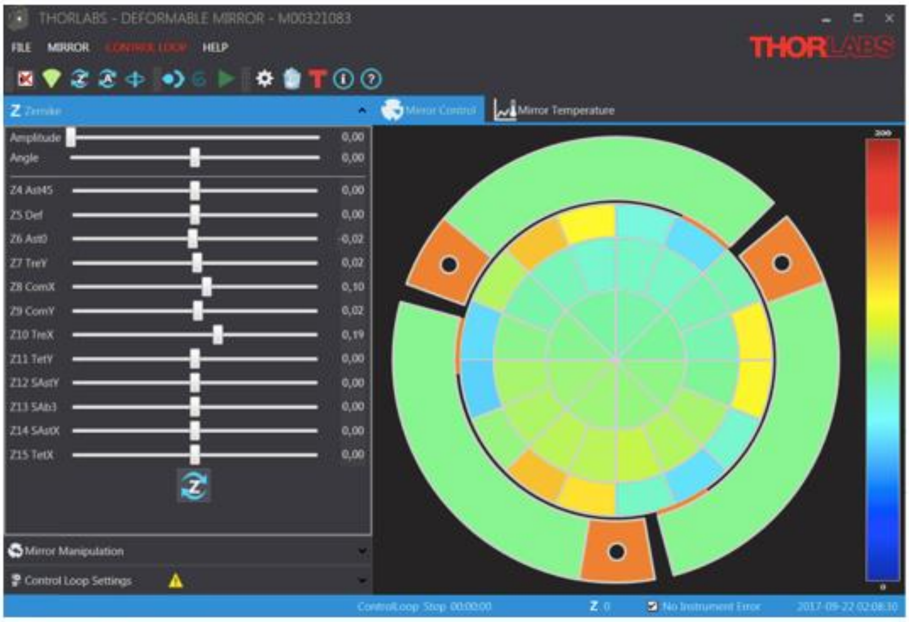

Supplementary Figure 1 : Optical system for 3D localization microscopy with deformable mirror

a Optical setup. The fluorescent signal comes from the right port of the microscope body, to which the camera is normally attached (top). The intermediate image plane (green segment) defines the original position of the camera chip. The beam is focused through a F=100 mm lens (F100), reflected by a silver mirror (Mirror) and reaches the surface of the deformable mirror (DM). The DM is placed on a XY translation stage, allowing to precisely center the mirror across the pupil image. The beam reflected by the DM then passes through a F=200 mm tube lens (F200) to form an image on the EMCCD camera (Camera). F100 and DM positions along the beam are precisely adjusted using a parallel laser beam and shear-plate interferometer. **b** Screenshot of the software that controls the DM. Sliders on the left panel allow to set Zernike coefficients. The colored panel on the right shows the voltages applied to the 40 actuators of the DM. The setting shown is typical for flat phase correction based on the images of fluorescent beads. See also Methods section "Optical setup and deformable mirror".

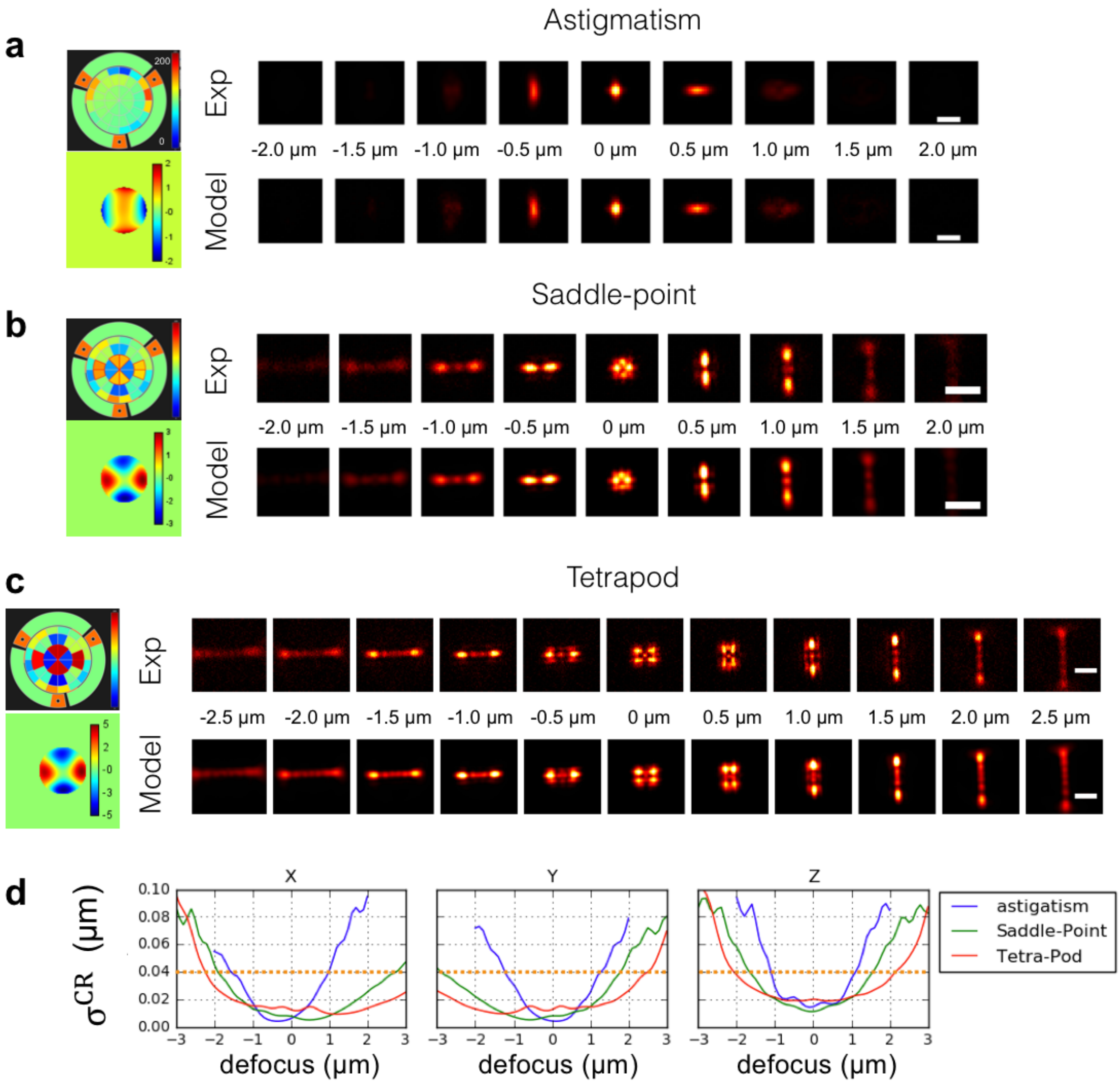

Supplementary Figure 2 : Engineered point spread functions

a-c Experimental and model PSFs obtained for three different settings of the deformable mirror (DM): astigmatism (**a**), saddle point (**b**) and tetrapod (**c**). The top left panel shows the voltages applied to the DM (as in Supplementary Fig. 1b). The top row (“Exp.”) shows a z-stack of a fluorescent bead (only 1 out of 5 images are shown), i.e. an approximation of the experimental PSF. The bottom left panel shows the phase model retrieved by ZOLA, in radians. The bottom row (“Model”) shows a z-stack of the model PSF. Scale bars are 1 μm for (**a**) and 2 μm for (**b-c**). **d** Theoretical localization precision limit σ^{CR} (obtained from the Cramér-Rao lower bound, or CRLB)[1,2] calculated by ZOLA for the three PSFs assuming 3,000 photons from the molecule and 20 background photons (as typical for our experiments) as function of defocus (distance of a bead from the focal plane), for the three coordinates x, y, and z (see Methods section "Theoretical limit to localization precision"). The saddle point PSF (green) outperforms the astigmatic PSF (blue) except in a very narrow axial range around 0 μm and achieves high localization precision (better than 40 nm, orange dashed line) over an axial range of ~3 μm instead of ~2 μm, in agreement with previous reports. The Tetrapod PSF (red) allows enhancing the axial range from ~3 μm to ~4 μm at the cost of slightly reduced localization precision around 0 μm.

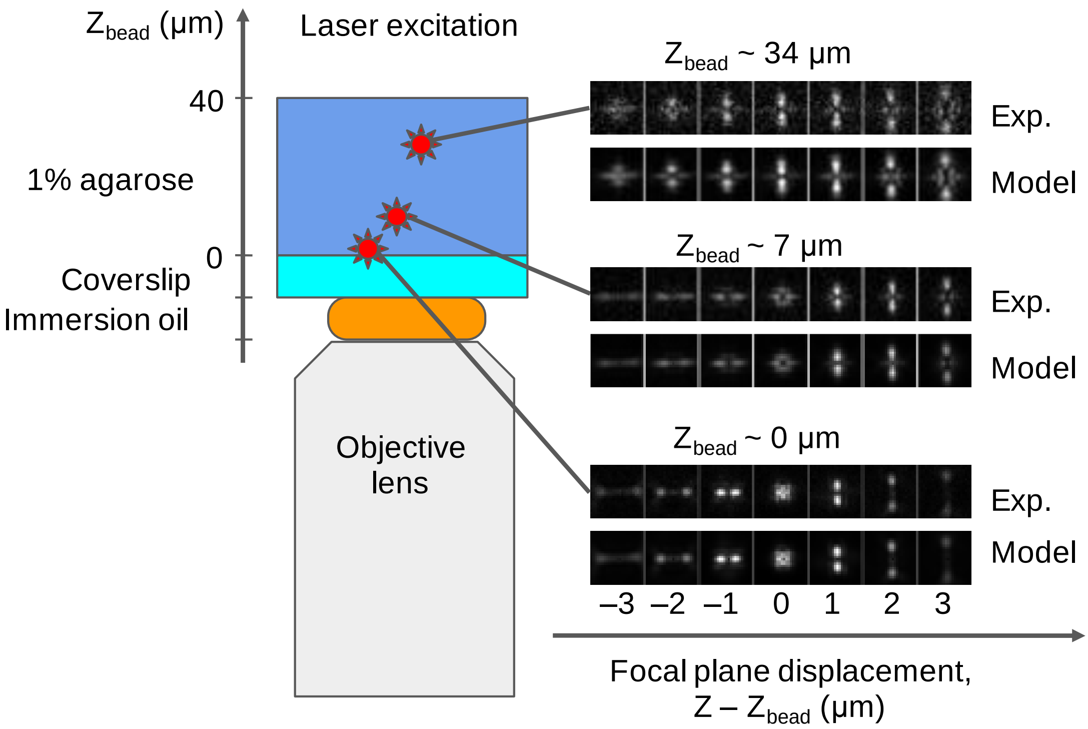

Supplementary Figure 3: ZOLA correctly models PSF aberrations deep inside index-mismatched medium

ZOLA's PSF modeling method can correctly account for deformations of the PSF due to a refractive index mismatch between the immersion and sample media. To demonstrate this, we imaged fluorescent beads suspended in an agarose gel (1% in PBS). The sample contains beads touching the coverslip and beads located at various distances from the coverslip. We configured the deformable mirror to create a saddle point PSF and acquired a z-stack of the sample by axially moving an oil immersion objective lens over 42 μm using 840 steps of 50 nm. From this z-stack we show three series of 7 slices separated by 1 μm, each corresponding to a distinct bead. The experimental image is labelled 'Exp.', the corresponding model PSF is labelled 'Model'. One bead was located deep inside the gel, at Z_{bead}~34 μm from the coverslip (top), another at Z_{bead}~7 μm from the coverslip (middle) (distances are as determined by the piezo displacement) and one was touching the coverslip (Z_{bead}~0 μm, bottom). The bead touching the coverslip shows an unaberrated saddle point PSF. However, deeper in the sample, the PSF is distorted by spherical aberrations, as seen for beads at Z_{bead}~7 and Z_{bead}~34 μm, which display quite different shapes from the bead at the coverslip. These aberrations are due to index mismatch between agarose (refractive index $n \approx 1.3$) and the coverslip with immersion medium ($n \approx 1.5$). Using only the images of beads at the coverslip for calibration, ZOLA nevertheless correctly predicts the shapes of the PSF at any depth inside the sample, as shown by the excellent agreement between the model images and the experimental images, even for the severe aberrations evident for Z_{bead}~34 μm.

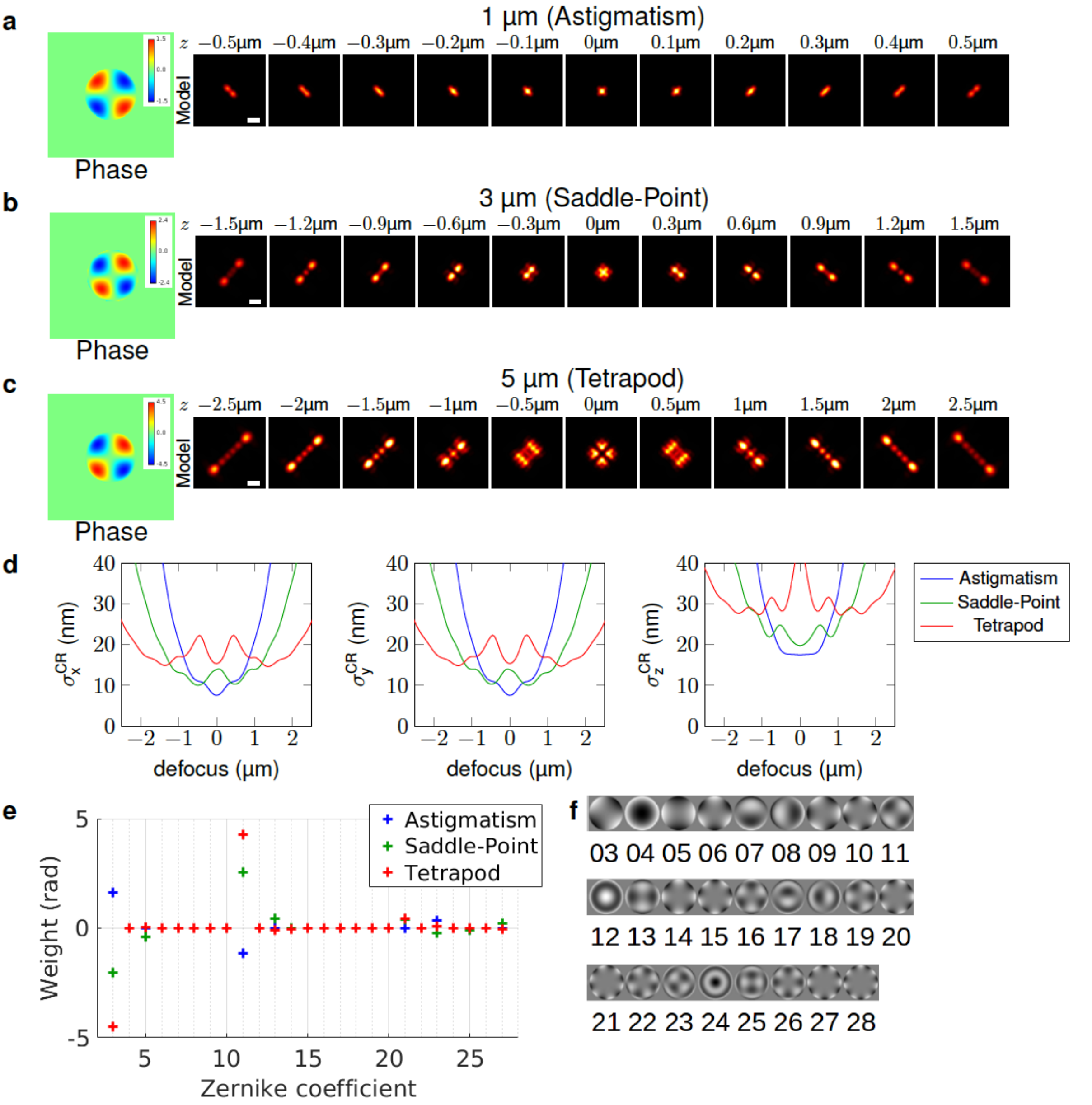

Supplementary Figure 4: PSF optimization by ZOLA

ZOLA can automatically compute the Zernike coefficients that achieve the best average axial localization precision for a specified axial range Δz. This is done by numerical optimization of the theoretical precision limit (CRLB)[1,2] of the z coordinate averaged over the axial range Δz (see Methods section "PSF optimization"). **a-c** Optimized phase and PSFs. The color map on the left shows the phase (in radians), the series of images on the right show z-sections of the corresponding model PSF. Panels (**a**), (**b**) and (**c**) correspond to optimized axial ranges Δz= 1, 3 and 5 μm, and result in astigmatic, saddle point and tetrapod PSFs, respectively. Scale bars, 1 μm. **d** The theoretical localization precision limit $\sigma^{CR} = \sqrt{\text{CRLB}}$ for the three coordinates (x,y and z) is plotted as function of defocus for the three PSFs (blue: astigmatism, green: saddle point, red: tetrapod). **e** The Zernike coefficients 3-28 (in radians) are shown for the three PSFs (blue: astigmatism, green: saddle point, red: tetrapod). **f** Zernike polynomials 3 to 28.

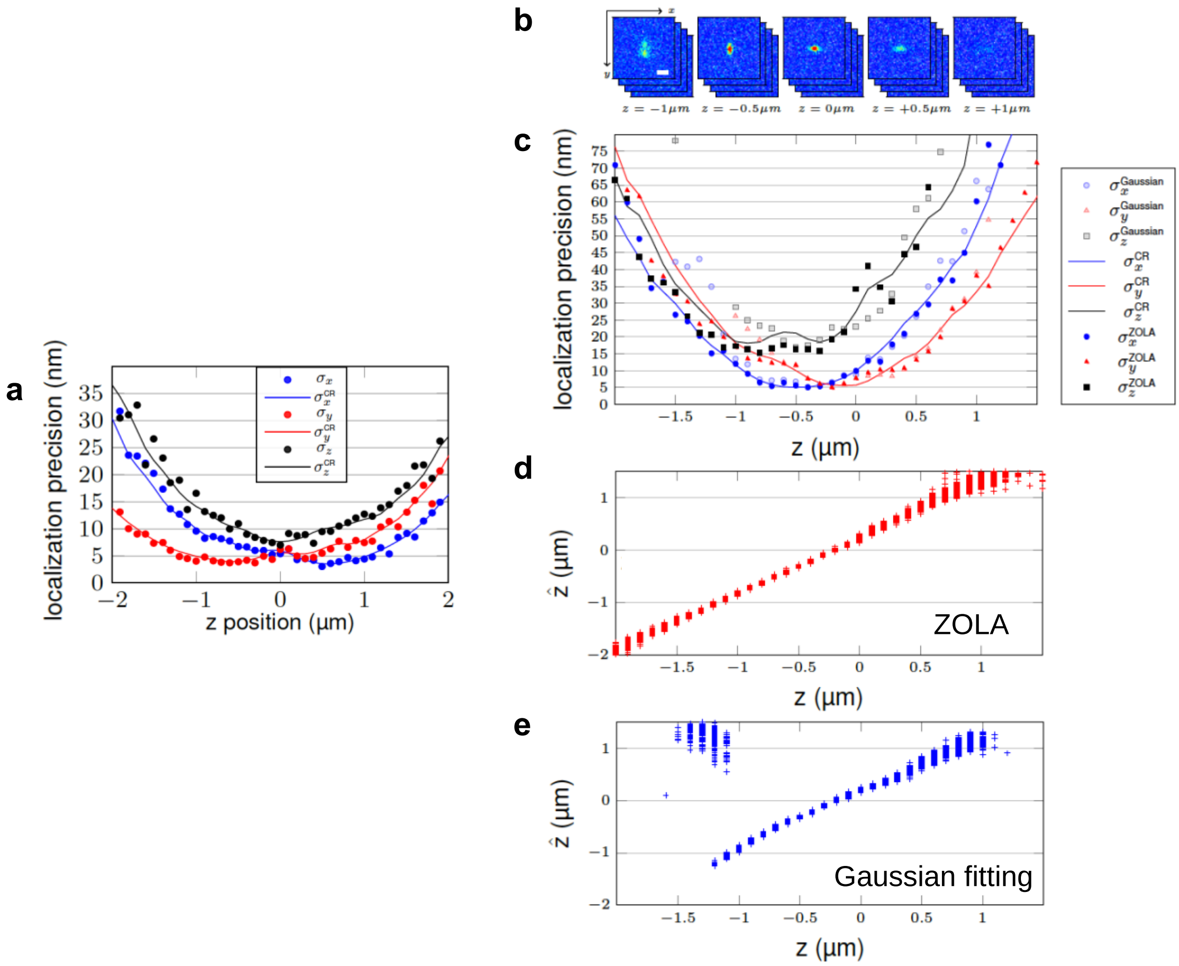

Supplementary Figure 5: Theoretical and empirical localization precision in simulations and for astigmatism

a Theoretical localization precision limit and ZOLA localization precision as assessed on simulated images, for a saddle point PSF and as function of axial coordinate z. This plot is similar to **Fig. 1f**, except that here the dots show the empirical localization precision (standard deviation of errors in the estimated coordinates x, y and z) computed on simulated single molecule images instead of experimental images. These images were simulated using the PSF obtained by phase retrieval from a fluorescent bead and assuming 5,000 signal photons and 10 background photons. **b-e** Localization precision assessment for an astigmatic PSF and comparison with Gaussian fitting. **b** Weakly excited fluorescent beads are imaged repeatedly (100 times) at different z positions with an astigmatic PSF and computationally localized in 3D using either ZOLA or the Gaussian fitting algorithm of ThunderSTORM[3]. Scale bar, 1 µm. **c** The empirical localization precision is measured as the standard deviation of calculated coordinates and plotted as symbols (dots, triangles and squares for x, y and z, respectively) for each axial position in the z-stack. Filled and shaded symbols refer to coordinates computed by ZOLA and Gaussian fitting, respectively. The solid curves are the theoretical limits to localization precision (CRLB[1]) computed by ZOLA for the average number of signal and background photons in the bead images. **d,e** Estimated axial coordinate of the bead ($\hat{z}$) plotted as function of the true z coordinate (as determined by the piezo), for ZOLA (**d**) and Gaussian fitting with ThunderSTORM[3] (**e**). Discrepancies reflect a bias in the localization algorithm. Panels (**c-e**) show that ZOLA allows optimal precision with little bias over a much larger axial range than Gaussian fitting.

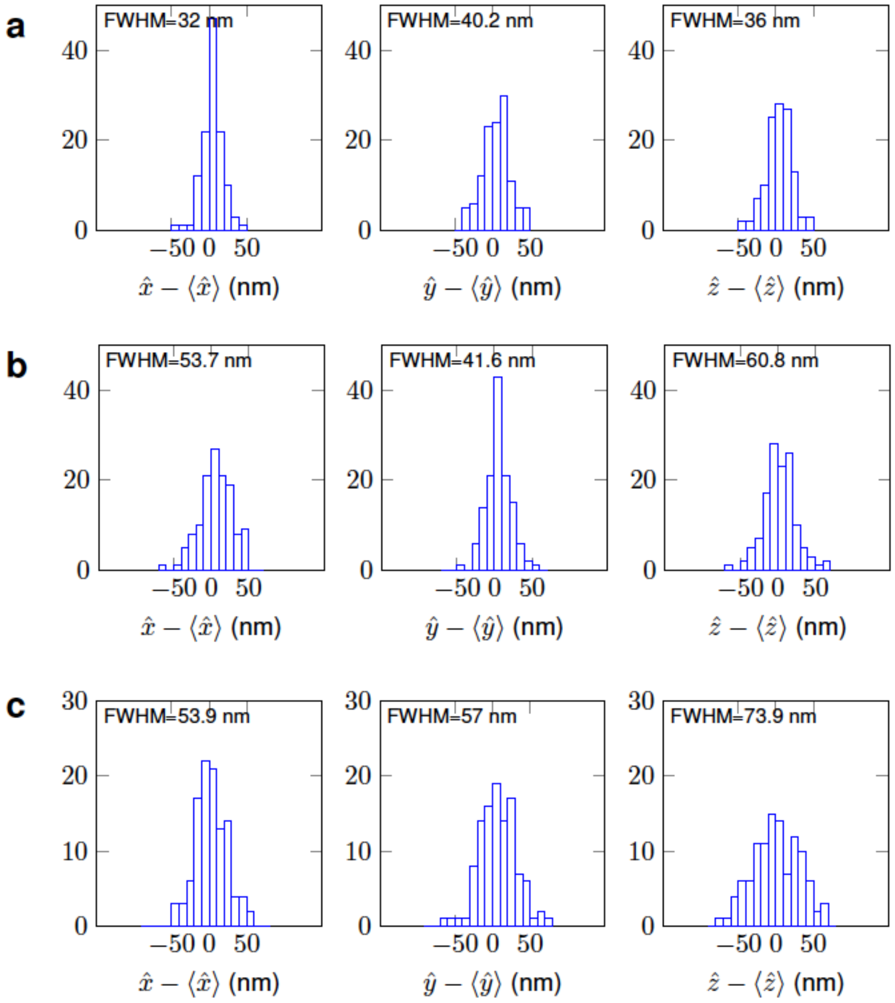

Supplementary Figure 6 : Resolution estimates from localization clusters

To estimate the localization precision in our 3D single molecule localization images, we manually selected isolated clusters of localizations, presumably originating from single fluorophores, and plot the histograms of the computed (estimated) coordinates $\hat{x} - \langle \hat{x} \rangle$, $\hat{y} - \langle \hat{y} \rangle$ and $\hat{z} - \langle \hat{z} \rangle$, relative to their average within each cluster. The FWHM (indicated in nm) along each axis is computed as 2.35 times the standard deviation of the corresponding coordinate and provides a measure of the expected resolution (ignoring sampling density[4]). Each row corresponds to a distinct image. **a** Resolution estimate for Fig. 1h, computed from n=122 localizations in 8 clusters. **b** Resolution estimate for Fig. 2a, computed from n=133 localizations in 7 clusters. **c** Resolution estimate for Fig. 2b, computed from n=114 localizations in 8 clusters.

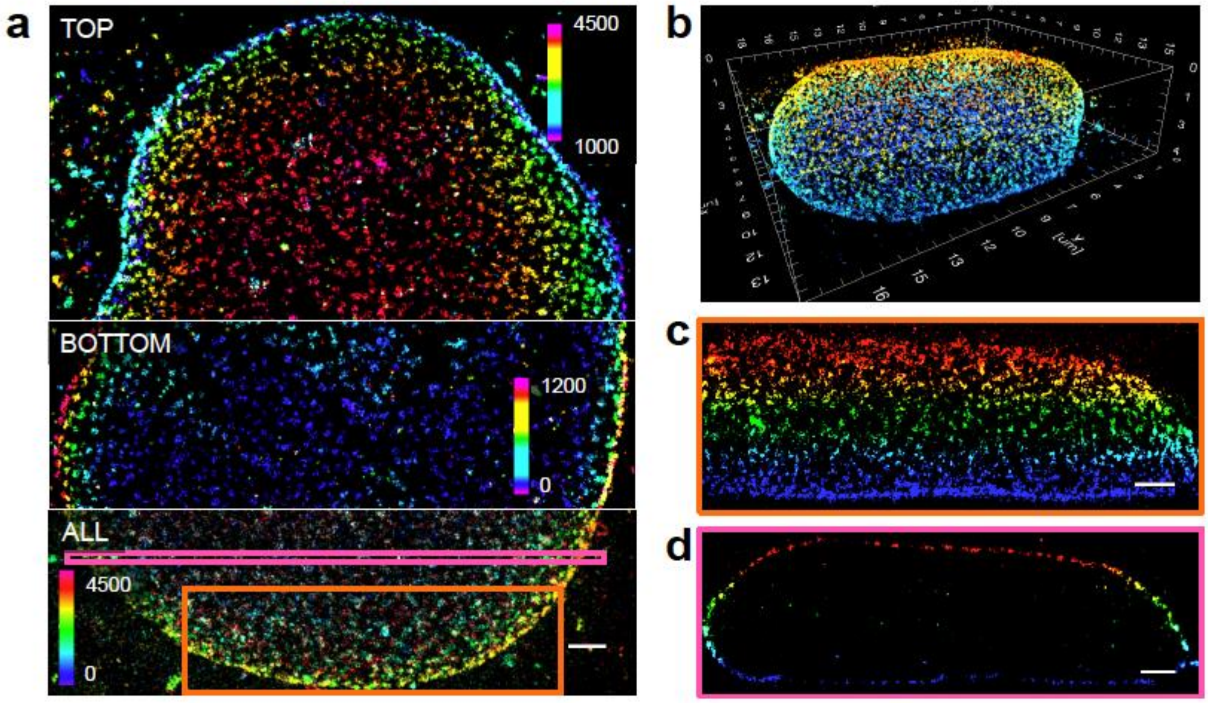

Supplementary Figure 7 : 3D super-resolution image of nuclear pores in a whole human nucleus

a 3D super-resolution image of the immunolabeled nucleoporin Nup133 in a HeLa cell. The image was reconstructed by ZOLA for a tetrapod PSF with water immersion objective (as in Fig. 2b). Color indicates axial coordinate z (see color bar, in nm). The portions labelled “TOP” and “BOTTOM” only show the top and bottom portions of the nucleus, respectively, whereas the portion labelled “ALL” shows the nucleus at all depths, as indicated by the three independent color bars (with units in nm). The axial range is 4.5 µm, allowing to visualize the entire nucleus as shown in the 3D perspective of (**b**). **c,d** (x,z) projection from the regions of interests enclosed by the orange and pink rectangles in (**a**), respectively. All scale bars are 1 µm.

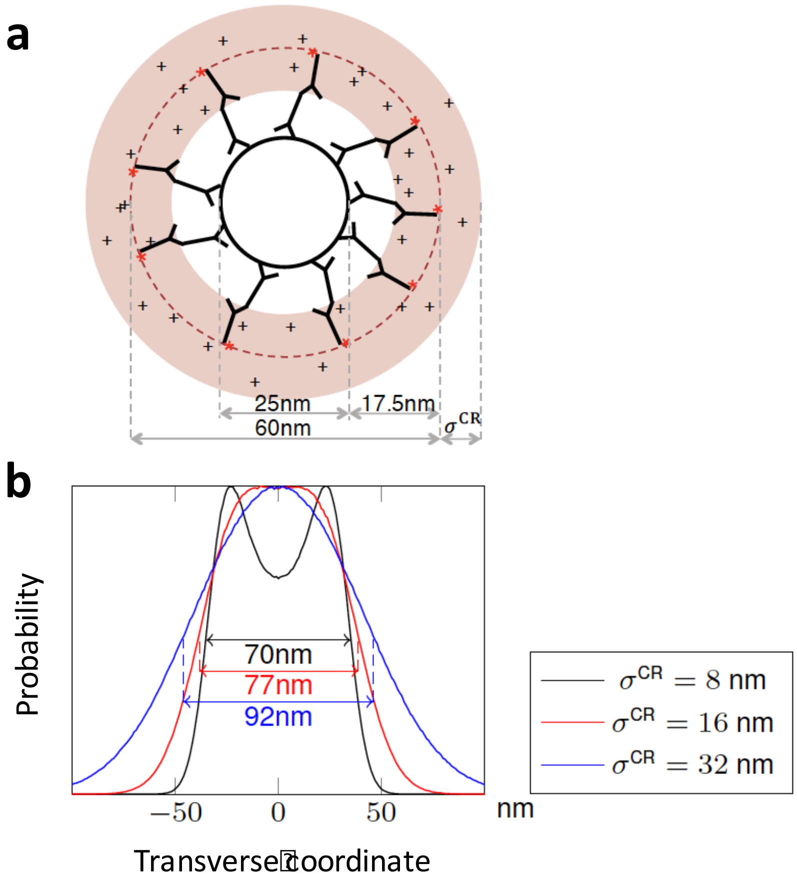

Supplementary Figure 8: Resolution and apparent width of immunolabeled microtubules

The apparent width of a microtubule filament in single molecule localization microscopy (SMLM) depends both on the localization precision and on the diameter of the fluorophore distribution around the microtubule center. **a** The schematic shows a cross section of a microtubule (small black circle of diameter 25 nm) labeled by primary and secondary antibodies (Y symbols). Because of the 17.5 nm size of this double antibody labeling, fluorophores (red stars) are expected to be at a ~30 nm distance from the microtubule center, i.e. on a cylinder of diameter ~60 nm (dashed red circle). Computational localizations of these fluorophores in SMLM (black crosses) are subject to random errors and therefore occupy a broader region (pale red ring). With an optimally precise localization algorithm, the standard deviation of these random errors is σ^{CR} as given by the CRLB[1] (see Methods section "Theoretical limit to localization precision"). **b** Simulated probability distribution of the computationally estimated coordinates across the microtubule for three assumed values of the localization precision σ^{CR} (8 nm, 16 nm, 32 nm, shown in black, red and blue, respectively). The full width at half maximum (FWHM) of these probability densities is indicated below arrows. Solid curves superposed to the experimental localization histograms in Fig. 2d,f were obtained in a similar fashion using the theoretical precision limits σ^{CR} computed by ZOLA for the average positions, signal and background photon counts corresponding to these localizations.

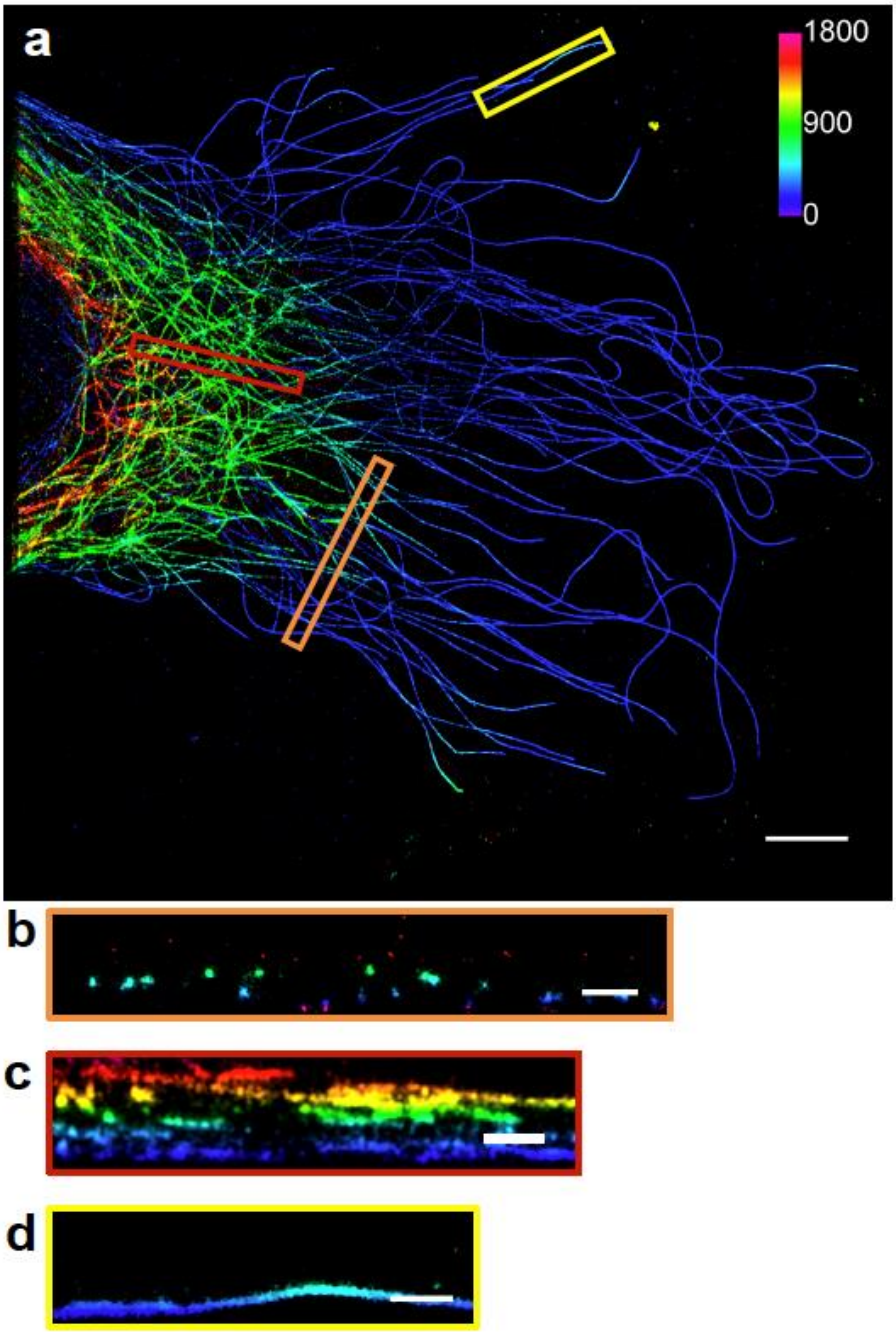

Supplementary Figure 9 : 3D super-resolution image of microtubules

a 3D super-resolution image of immunolabeled microtubules in a U-373 MG cell reconstructed by ZOLA for a saddle point PSF (as in Fig. 2e). The color encodes the axial coordinate z (see color bar, in nm). Scale bar, 5 μm. **b-d** Side views show projections of the regions enclosed by orange, red and yellow rectangles, respectively, along the axis perpendicular to the long side of each rectangle. Scale bars, 1 μm.

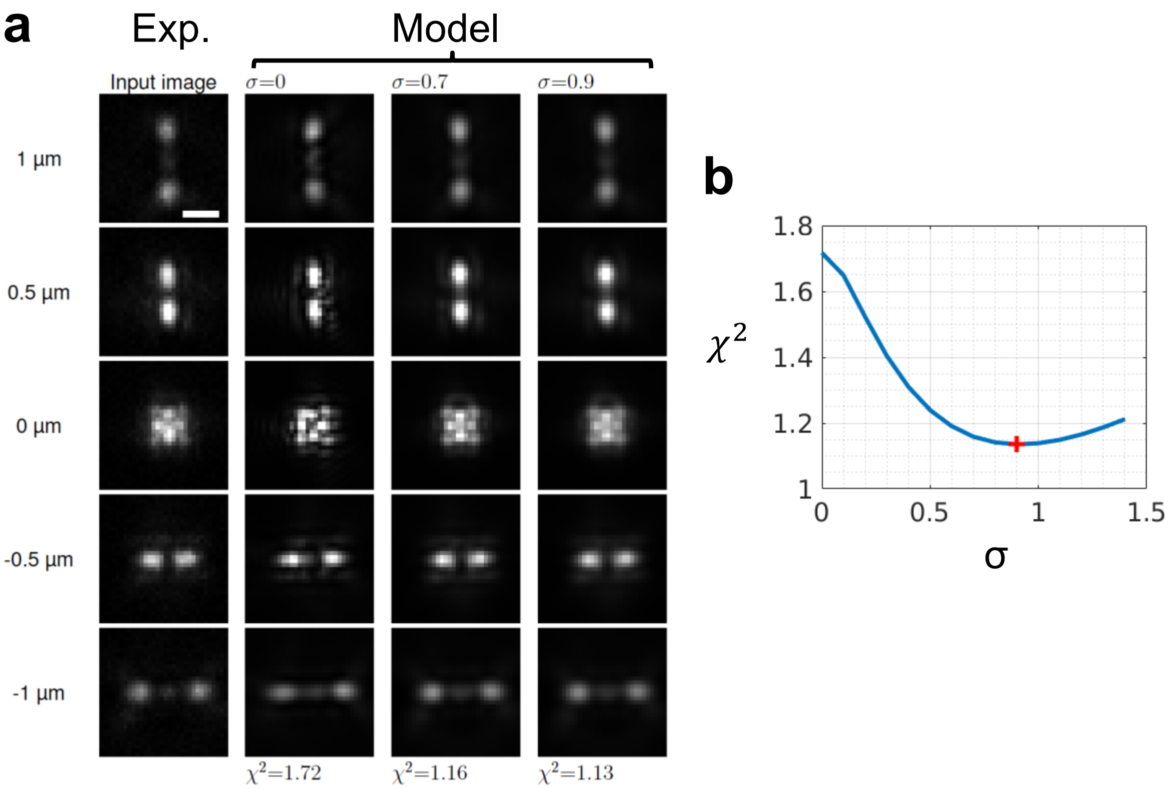

Supplementary Figure 10 : Effect of Gaussian convolutional kernel on PSF model

The PSF model used by ZOLA involves a convolution with a Gaussian kernel of standard deviation σ (Methods section "Image formation model", Equation (4)). This kernel was introduced to improve the match between the model and the data as shown in this figure. **a** The first column from the left ('Exp.') shows a z-stack of a fluorescent bead imaged with a saddle point PSF, for z=1 µm, 0.5 µm, 0 µm, -0.5 µm and -1 µm. The 2nd, 3rd and 4th columns ('Model') show the PSF model generated by ZOLA with σ=0, σ=0.7 and σ=0.9, respectively. The value σ=0.9 was obtained by maximum likelihood estimation of the PSF model, treating σ as a parameter to be estimated along with the Zernike coefficients. Note how the model with σ=0 displays sharper features than visible in the experimental data, and that models with σ=0.7 or σ=0.9 provide a much better match to the data. This is confirmed by the χ^2 value of the residual between model and data shown on the bottom. Scale bar, 1 µm. **b** The χ^2 of the residual between the model and the image is plotted for different values of σ, exhibiting a minimum at σ=0.9.

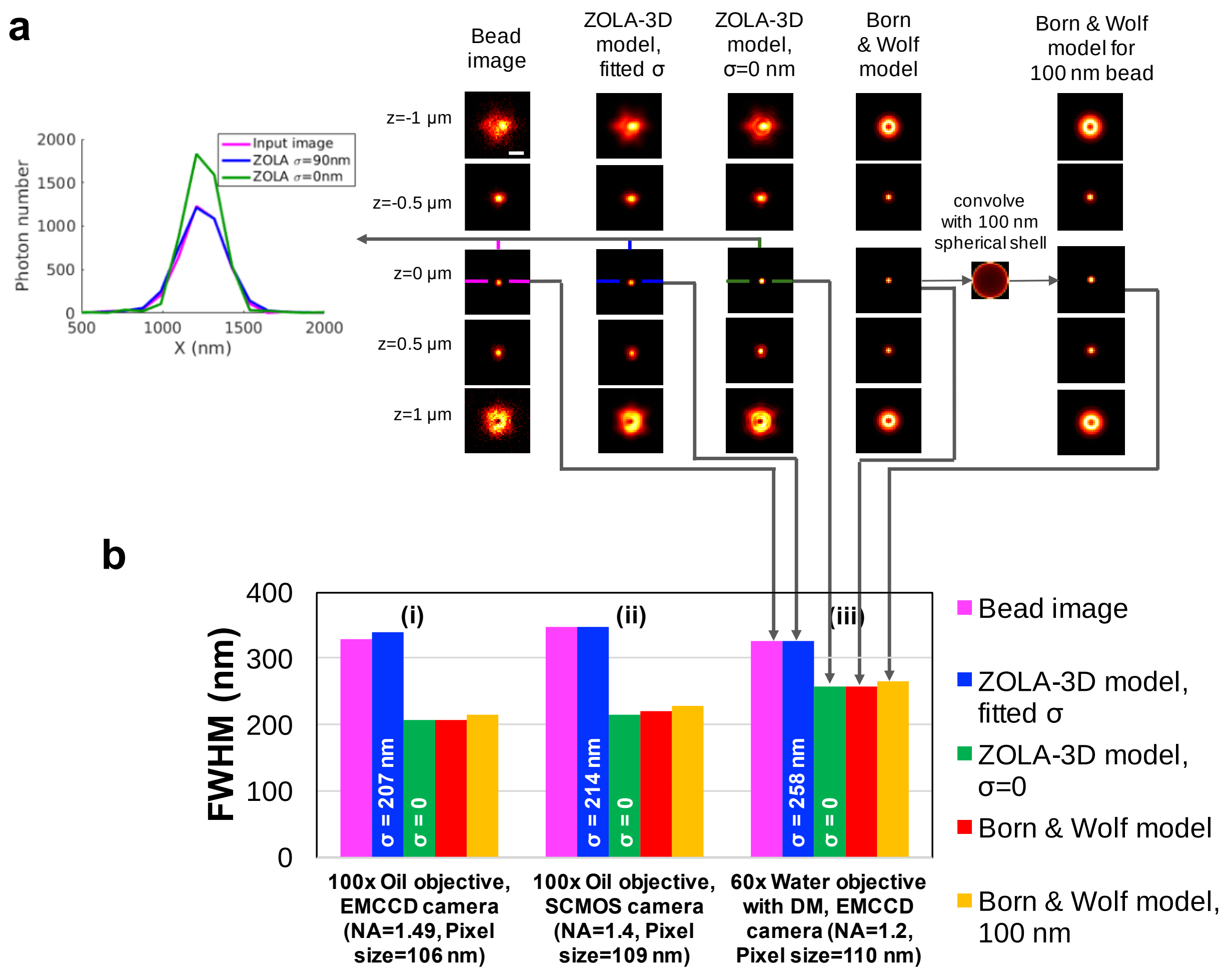

Supplementary Figure 11: Robustness of ZOLA PSF modeling on different microscopes

To test the robustness of PSF modeling by ZOLA, calibration z-stacks of fluorescent beads were acquired on three different microscopes: (i) a microscope equipped with an oil immersion TIRF objective (Nikon Apo TIRF; numerical aperture NA=1.49, immersion medium refractive index n=1.518) and an EMCCD camera (pixel size p=106 nm), (ii) a microscope equipped with a 100x oil immersion objective (Nikon Plan Apo λ; NA=1.45, n=1.518) and an sCMOS camera (p=109 nm), and (iii) a microscope equipped with a 60x water immersion objective (Nikon Plan Apo VC; NA=1.2, n=1.33), an EMCCD camera (p=110 nm) and a deformable mirror set to flat position. **a** The right part of the panel shows experimental images and model PSFs for the microscope (iii) at selected z coordinates. The left column shows the image of a fluorescent 100 nm diameter bead. The second column shows the model retrieved by ZOLA with a fitted standard deviation σ of the Gaussian blurring kernel. The third columns shows the model retrieved by ZOLA without Gaussian blurring (i.e. σ=0). The fourth column shows the diffraction limited PSF predicted by the Born & Wolf model in absence of aberrations, as generated by the 'PSF generator' plugin of ImageJ[5]; the fifth column shows this PSF convolved with a spherical shell of diameter 100 nm to account for the finite size of the fluorescent bead. Scale bar, 1 μm. The plot on the left shows intensity profiles across the horizontal line passing through the bead image and the models retrieved by ZOLA for z=0 μm. Note that ZOLA's PSF model matches the experimental bead image data well when σ is fitted, but not when σ=0. **b** The full width at half maximum (FWHM) of intensity profiles through the experimental bead image or the PSF models is shown for the three microscopes. The FWHM are computed from Gaussian fits to each profile using ThunderSTORM[3]. Note that for the three microscopes tested, the FWHM measured on the bead images (pink) are different from each other and significantly larger than predicted by the Born & Wolf model (red); this cannot be explained by the 100 nm diameter of the bead, which increases the FWHM only very slightly (orange). In all three cases, the FWHM of the model PSF retrieved by ZOLA matches the experimental data well when σ is fitted (blue), but not for σ=0 (green). The optimal σ values depend on each microscope: σ=207.3 nm, 214 nm and 258 nm for microscopes (i), (ii) and (iii), respectively.

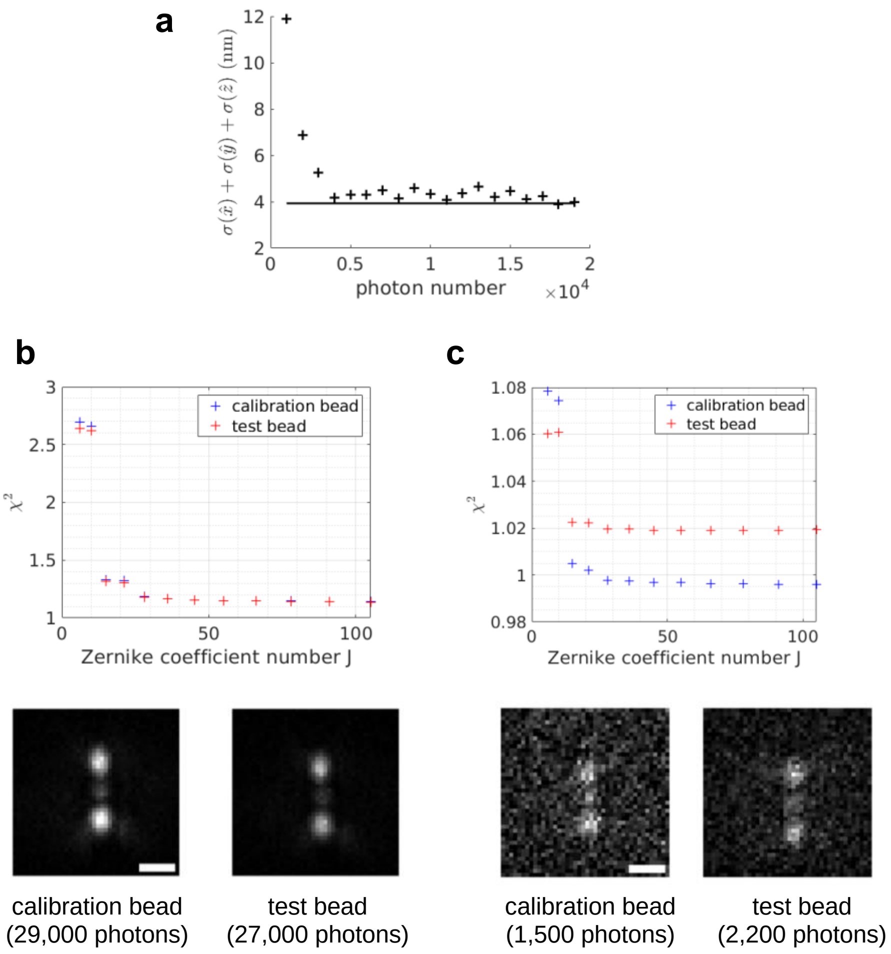

Supplementary Figure 12: Bead photon counts and Zernike coefficients required for accurate PSF calibration

a To evaluate the bead photon counts required for precise calibration of the PSF, we first defined a reference PSF (by performing PSF calibration on experimental images of a fluorescent bead with a saddle point PSF), then used this reference PSF to simulate z-stacks of a fluorescent bead (over a 6 μm axial range with steps of 50 nm) with a variable number of photons, from n_{bead}=1,000 to n_{bead}=19,000 and a background of 2 photons. We then applied phase retrieval on each of these z-stacks, thereby obtaining 19 distinct model PSFs. Next, we used simulated single molecule images with n=100,000 photons (uniformly distributed over a 4 μm axial range) to evaluate the localization precision of ZOLA for each of these PSF models. The crosses show the localization precision (standard deviation of localization errors) summed over the three coordinates, as function of the simulated bead photon counts n_{bead}. The horizontal line shows the precision obtained with ZOLA when directly using the reference model, i.e. for perfect PSF calibration. Note that with n_{bead}=4,000 photons or more, the precision is no longer limited by PSF modeling errors, suggesting that PSF calibration is optimal for beads with n_{bead}>4,000 photons (and a background of 2 photons). **b,c** To evaluate the number of Zernike coefficients J required for accurate PSF calibration, we compared fluorescent bead images to the model PSF for high (**b**) and low (**c**) numbers of photons. We use a single bead ('calibration bead') for PSF calibration and use a distinct bead ('test bead') for validation. The number of photons is indicated. We vary the number of Zernike coefficients J from 15 to 105 (ten values in total) and apply ZOLA's phase retrieval feature to the calibration bead image to model the PSF for each J. The χ^2 residual between the image and the model is shown for both the calibration image (blue crosses) and the test image (red crosses). For high photon numbers (**b**), the residual decreases monotonously with J for both the calibration and test images, up to J >100. For low photon numbers (**c**), the residual decreases monotonously for the calibration bead image, but very slightly increases for the test image when J ≥78, indicating minor overfitting. In practice, with n_{bead}≥5,000, a single bead and J ≥28 Zernike coefficients are sufficient to yield a χ^2 residual close to the optimum. For beads with lower photon counts or overlapping PSFs, we recommend using multiple beads for PSF calibration. Scale bars, 1 μm.

Supplementary references:

1. Ober, R. J., Ram, S. & Ward, E. S. Localization accuracy in single-molecule microscopy. *Biophys J* **86,** 1185–1200 (2004).
2. Shechtman, Y., Sahl, S. J., Backer, A. S. & Moerner, W. E. Optimal Point Spread Function Design for 3D Imaging. *Phys. Rev. Lett.* **113,** 133902 (2014).
3. Ovesny, M., Kiek, P., Borkovec, J., Vindrych, Z. & Hagen, G. M. ThunderSTORM: a comprehensive ImageJ plug-in for PALM and STORM data analysis and super-resolution imaging. *Bioinformatics* **30,** 2389–2390 (2014).
4. Legant, W. R. *et al.* High-density three-dimensional localization microscopy across large volumes. *Nat. Methods* **13,** 359–365 (2016).
5. Kirshner, H., Sage, D. & Unser, M. 3D PSF Models for Fluorescence Microscopy in ImageJ. in *Proceedings of the Twelfth International Conference on Methods and Applications of Fluorescence Spectroscopy, Imaging and Probes (MAF'11)* **154,** (2011).
