# Supplementary material for: ZOLA-3D allows flexible 3D localization microscopy over an adjustable axial range
Source: Nat Commun. 2018 Jun 19;9:2409. doi: 10.1038/s41467-018-04709-4 (PMC6008307; doi:10.1038/s41467-018-04709-4)
Supplement: Supplementary file 2 — Description of Additional Supplementary Files [file 41467_2018_4709_MOESM2_ESM.pdf]

Description of Additional Supplementary Files

File Name: Supplementary Movie 1
Description: This movie shows 51 consecutive frames from the sequence of low resolution images used to reconstruct the 3D super-resolution image of mitochondria shown in Figure 1h. Single molecules are visible as saddle point PSFs.

File Name: Supplementary Movie 2
Description: This movie shows an animated view of the 3D super-resolution image of mitochondria shown in Figure 1h.

File Name: Supplementary Movie 3
Description: This movie shows an animated view of the 3D super-resolution image of nuclear pores shown in Figure 2b.

File Name: Supplementary Movie 4
Description: This movie shows how the PSF can be changed during image acquisition to adapt to the decreasing density of activated molecules. The left panel shows the sequential acquisition of single molecule images, first with an astigmatic PSF (see Figure 2c), then with a saddle point PSF (see Figure 2e) (only one out of every 500 frames is shown to reduce movie size). The right panel shows the progressive 3D super-resolution image reconstruction. Note how that the axial range of the super-resolution image is 600 nm for the astigmatic PSF and 2.5 µm for the saddle point PSF. Scale bar, 10 µm.
