# Supplementary figures and images for: ZOLA-3D allows flexible 3D localization microscopy over an adjustable axial range
Source: Nat Commun. 2018 Jun 19;9:2409. doi: 10.1038/s41467-018-04709-4 (PMC6008307; doi:10.1038/s41467-018-04709-4)

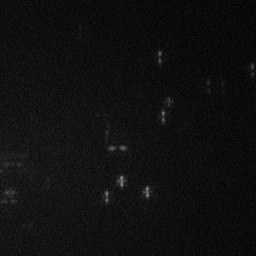

Supplement: Supplementary file 3 — Supplementary Movie 1 [file 41467_2018_4709_MOESM3_ESM.gif]

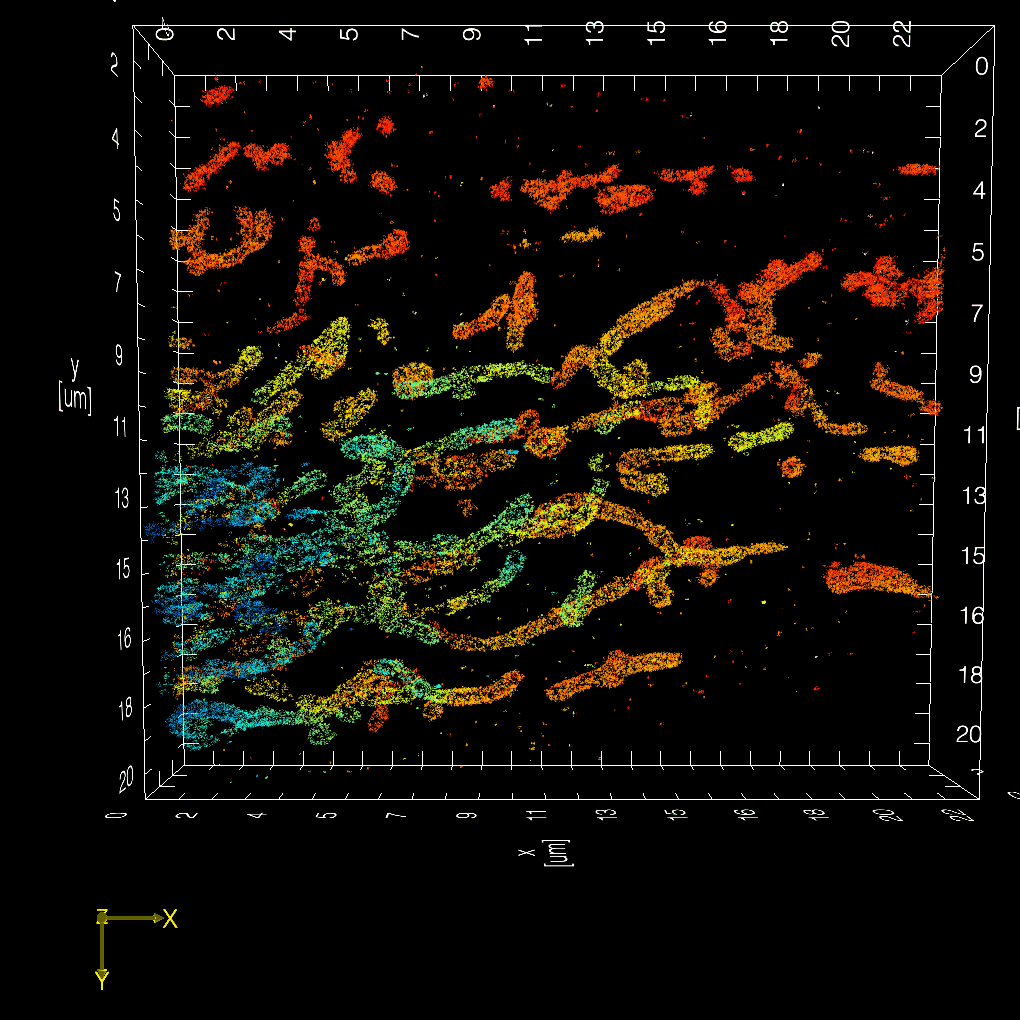

Supplement: Supplementary file 4 — Supplementary Movie 2 [file 41467_2018_4709_MOESM4_ESM.gif]

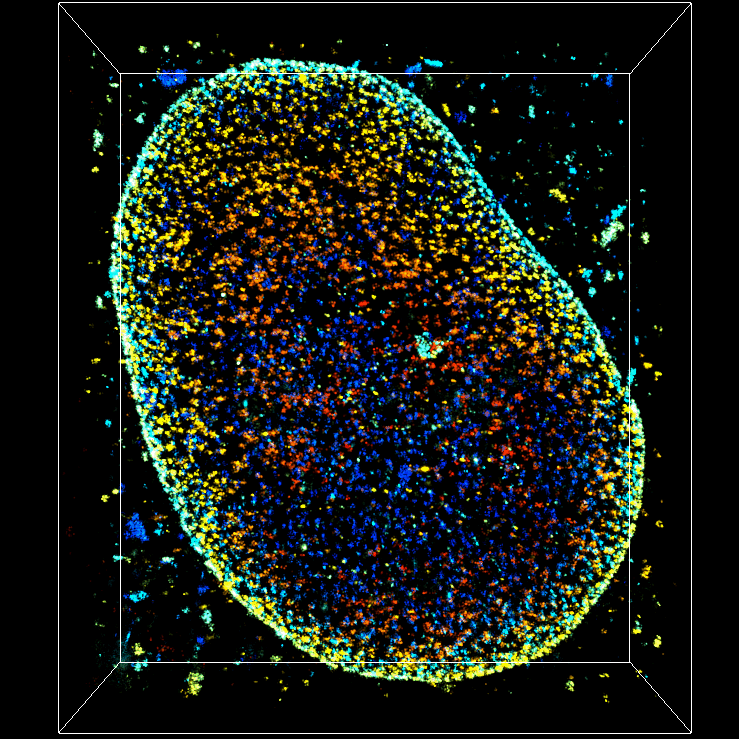

Supplement: Supplementary file 5 — Supplementary Movie 3 [file 41467_2018_4709_MOESM5_ESM.gif]

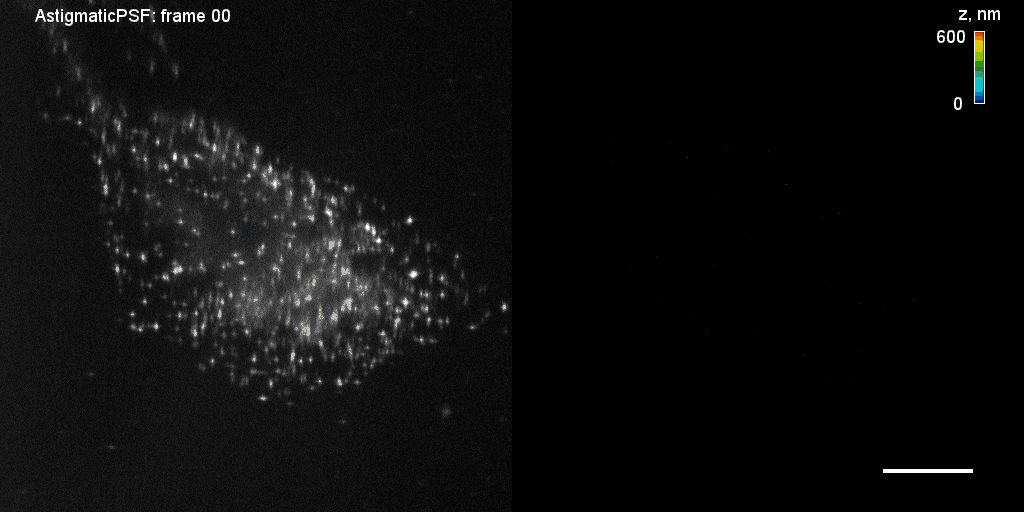

Supplement: Supplementary file 6 — Supplementary Movie 4 [file 41467_2018_4709_MOESM6_ESM.gif]
